# Supplementary material for: Discovery of isoflavone phytoalexins in wheat reveals an alternative route to isoflavonoid biosynthesis
Source: Nat Commun. 2023 Nov 1;14:6977. doi: 10.1038/s41467-023-42464-3 (PMC10620232; doi:10.1038/s41467-023-42464-3)
Supplement: Supplementary file 1 — Supplementary Information [file 41467_2023_42464_MOESM1_ESM.pdf]

Supplementary Information for:

Discovery of Isoflavone Phytoalexins in Wheat Reveals an Alternative Route to Isoflavonoid Biosynthesis

Guy Polturak[*‡1,2], Rajesh Chandra Misra[‡1], Amr El-Demerdash[1,3], Charlotte Owen[1], Andrew Steed[4], Hannah P McDonald[5], JiaoJiao Wang[1,6], Gerhard Saalbach[7], Carlo Martins[7], Laetitia Chartrain[4], Barrie Wilkinson[5], Paul Nicholson[4], Anne Osbourn[*1]

[1]Biochemistry and Metabolism Department, John Innes Centre, Norwich NR4 7UH, United Kingdom

[2]Institute of Plant Sciences and Genetics in Agriculture, The Hebrew University of Jerusalem, Rehovot 7610001, Israel

[3]Division of Organic Chemistry, Department of Chemistry, School of Sciences, Mansoura University, Mansoura-35516, Egypt

[4]Crop Genetics Department, John Innes Centre, Norwich NR4 7UH, United Kingdom

[5]Molecular Microbiology Department, John Innes Centre, Norwich NR4 7UH, United Kingdom

[6]Tsinghua-Peking Joint Center for Life Sciences, and School of Life Sciences, Tsinghua University, Beijing 100084, China

[7]Proteomics Facility, John Innes Centre, Norwich NR4 7UH, United Kingdom

[‡]These authors contributed equally to this work

[*]To whom correspondence should be addressed.

Emails: anne.osbourn@jic.ac.uk; guy.polturak@mail.huji.ac.il

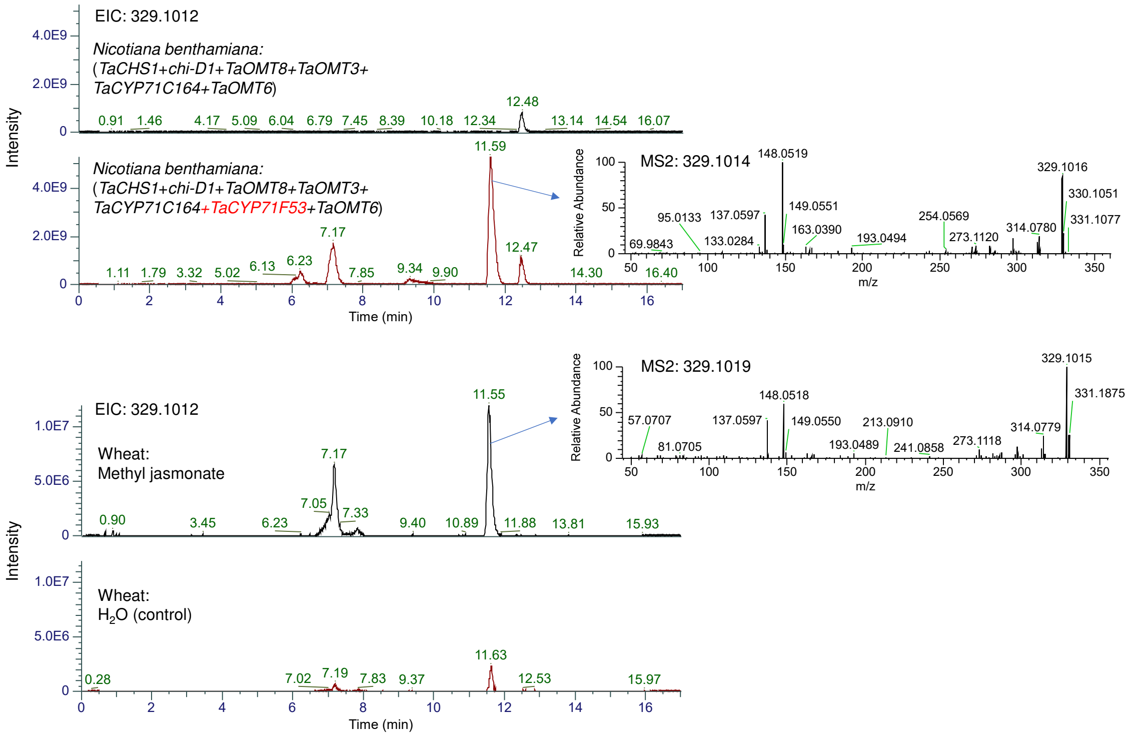

Supplementary Fig. 1. Extracted ion chromatograms and MS2 fragmentation of (*m/z* 329.1012) ion in wheat and *Nicotiana benthamiana*. Top chromatograms, extract from *N. benthamiana* leaf infiltrated with BGC4(5D) genes (*TaCHS1*, *chi-D1*, *TaOMT8*, *TaOMT3*, *TaCYP71C164*, *TaCYP71F53*, *TaOMT6*), or with all genes excluding *TaCYP71F53* (control). Bottom chromatograms, extracts from methyl jasmonate-treated wheat blades (cv. Chinese Spring), or from control (water treated-blades).

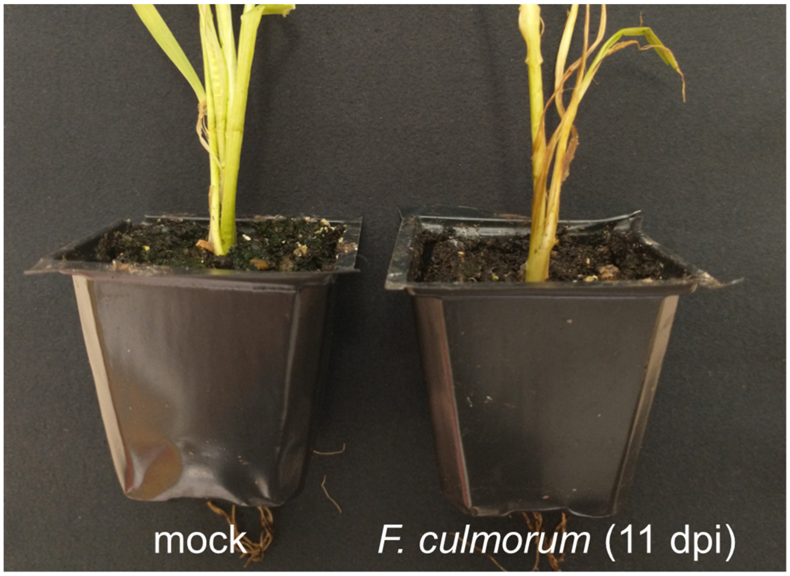

Supplementary Fig. 2. 'Chinese Spring' wheat plants infected with *F. culmorum* (isolate FC2021) or mock-treated. Image taken 11 days post infection.

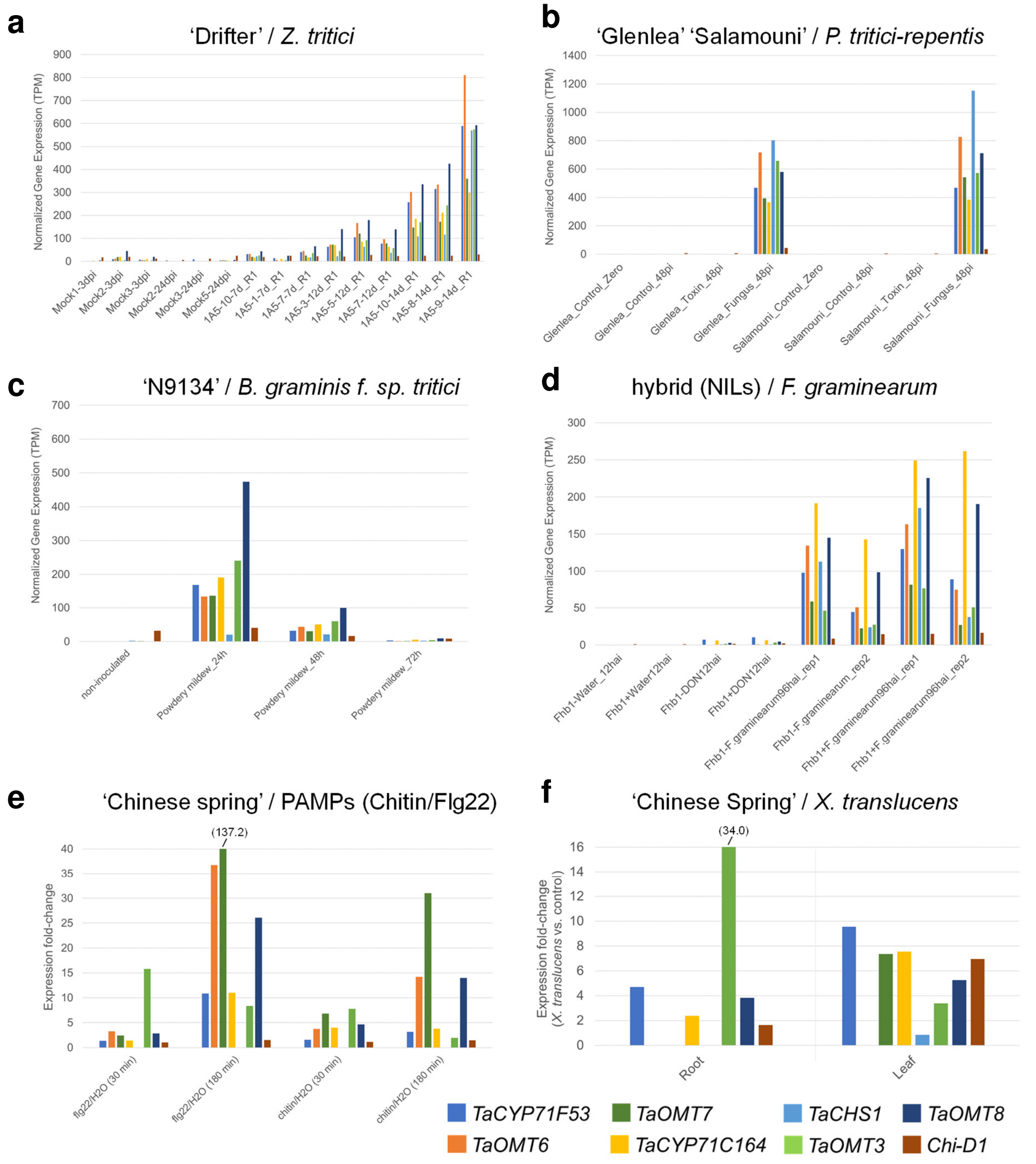

Supplementary Fig. 3. Gene expression data of BGC4(5D) genes in six biotic stress experiments. Normalized values (transcripts per million) (A-D) or expression fold-change (E-F) is shown, derived from RNA-seq data at Wheatomics 1.0[1] (http://wheatomics.sdau.edu.cn/). **a**, infection of wheat cv. 'Drifter' with *Zymoseptoria tritici*[2]. **b**, infection of wheat varieties 'Glenlea' and 'Salamouni' with fungal pathogen *Pyrenophora tritici-repentis* or infiltration with the toxin Ptr ToxA (https://www.ncbi.nlm.nih.gov/bioproject/PRJNA327829). **c**, infection of wheat line N9134 with powdery mildew (*Blumeria graminis* f. sp. *tritici*; *Bgt*)[3]. **d**, infection with *Fusarium graminearum* of wheat near isogenic lines that are resistant (Fhb1+) or susceptible (Fhb1-) to Fusarium head blight[4]. **e**, elicitation of cv. 'Chinese Spring' plants with PAMPs chitin or Flg22[5]. Fold-change of TPM values (treatment vs. control) is shown. **f**, infection of cv. 'Chinese Spring' plants with bacterial pathogen *Xanthomonas translucens*[6]. Fold-change of TPM values (expression following infection with *X. translucens* vs. control, in root or leaf tissue) is shown. Fold-change values are shown in brackets, where column exceeds Y-axis view.

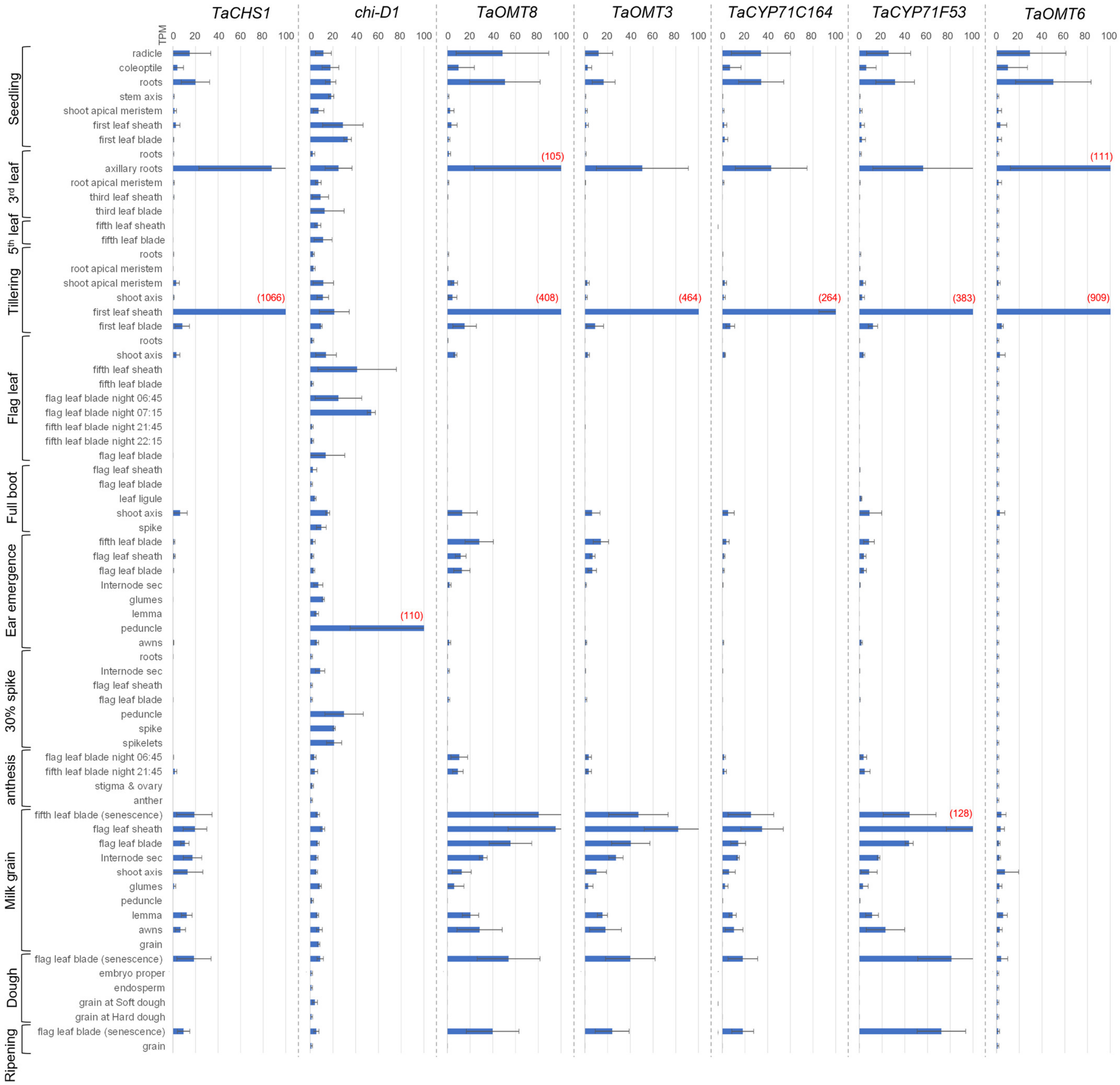

Supplementary Fig. 4. Expression patterns of BGC4(5D) genes across tissues and developmental stages. Transcript per million (TPM) values are shown for BGC4(5D) genes, in a developmental/time course RNA-seq analysis of wheat *cv. Azhurnaya*[5]. Expression data was extracted from Wheatomics 1.0[1] (http://wheatomics.sdau.edu.cn/) and wheat eFP browser[7] (https://bar.utoronto.ca/efp_wheat/cgi-bin/efpWeb.cgi). TPM values are shown (in red, brackets) where they exceed the given scale.

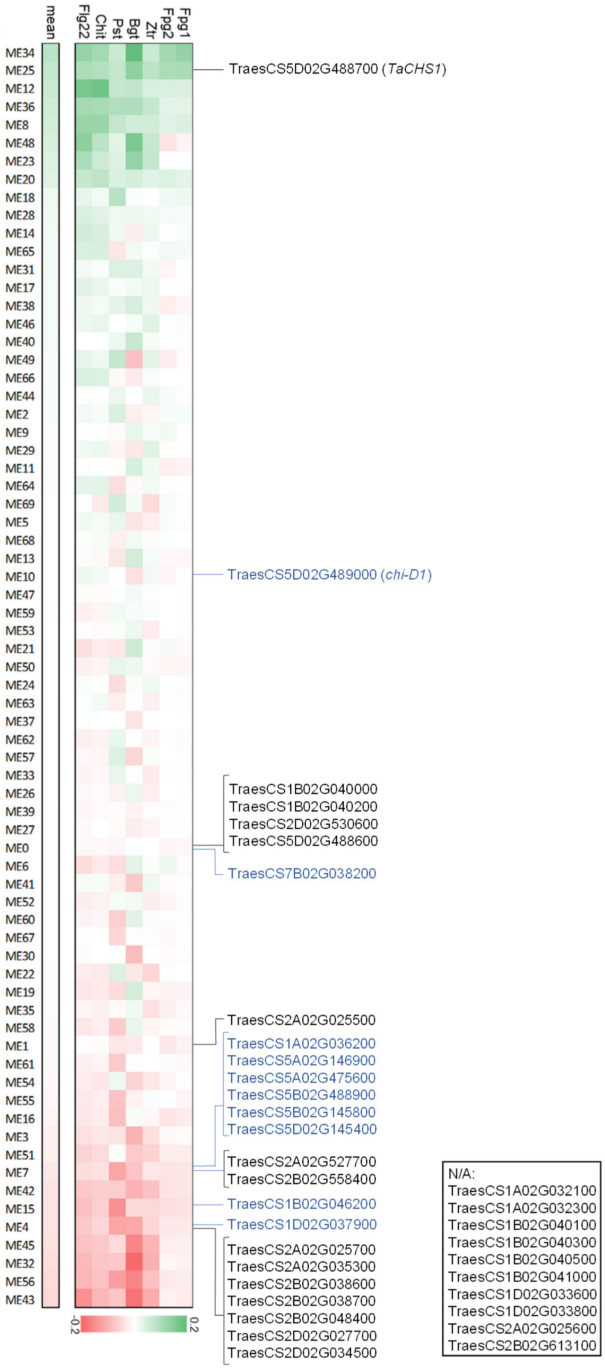

Supplementary Fig. 5. Assignment of putative wheat *CHS* and *CHI* genes to gene expression modules of a biotic stress related WGCNA. 69 gene expression modules are ordered from the most highly upregulated (top) to the most highly downregulated (bottom), following pathogen infection or treatment with chitin or flagellin. 25 Putative *CHS* genes (in black) and 10 putative *CHI* genes (in blue) were assigned to the different modules. N/A denotes unassigned (non-expressed) genes. Heatmap image adopted from Polturak et al.,[8]. Fpg1, *Fusarium pseudograminearum*; Fpg2, *Fusarium pseudograminearum*; Ztr, *Zymoseptoria tritici*; Bgt, *Blumeria graminis f. sp. tritici*; Pst, *Puccinia striiformis f. sp. Tritici*; Chit, chitin; Flg22, flagellin peptide.

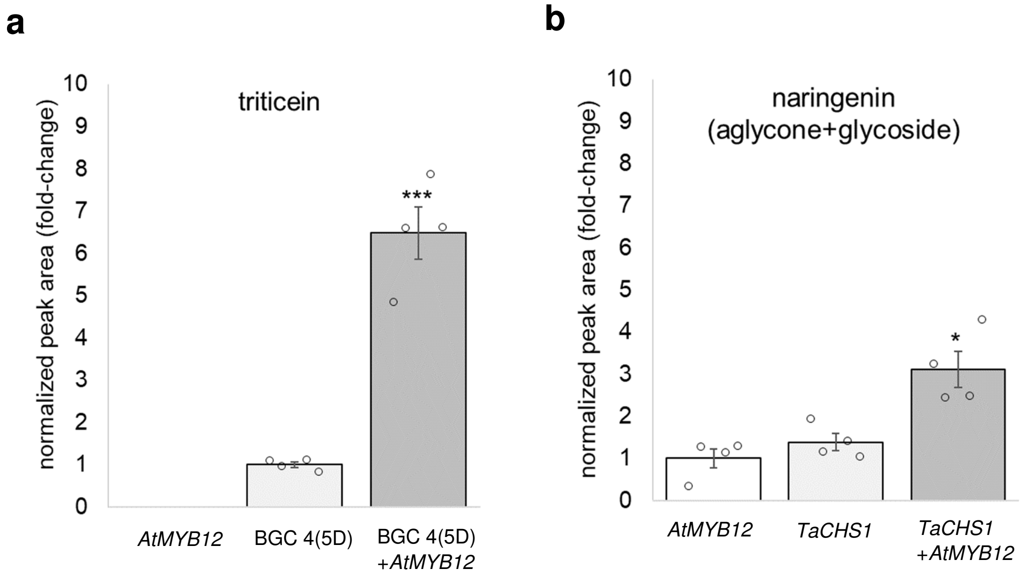

Supplementary Fig. 6. Relative abundance of triticein and naringenin in agroinfiltrated *Nicotiana benthamiana* leaves. a, relative abundance of triticein (**6**) in *N. benthamiana* leaves infiltrated with agrobacteria strains carrying expression vectors for *AtMYB12* only, BGC4(5D) genes (*TaCHS1*, *chi-D1*, *TaOMT8*, *TaOMT3*, *TaCYP71C164*, *TaCYP71F53*, *TaOMT6*), or co-infiltration of *AtMYB12* together with BGC(5D) genes. **b**, relative abundance of naringenin (**1**) in *N. benthamiana* leaves infiltrated with agrobacteria strains carrying expression vectors for *AtMYB12*, *TaCHS1*, or co-infiltration of both. Normalized peak area of naringenin is the sum of normalized peak areas of the aglycone ion (*m/z* 273.0752) and the ion of the single glycosylated compound (*m/z* 435.1274). Relative quantification values (in fold-change) indicate means of three biological replicates ± SEM. Asterisks denote t-test statistical significance of differential expression. *, *p*-val<0.05. ***, *p*-val<0.001. Exact *p*-values: $1.21 \cdot 10^{-4}$ (panel **a**), 0.0112 (panel **b**). For panels **a**,**b**, n=4 (biological replicates). Source data are provided as a Source Data file.

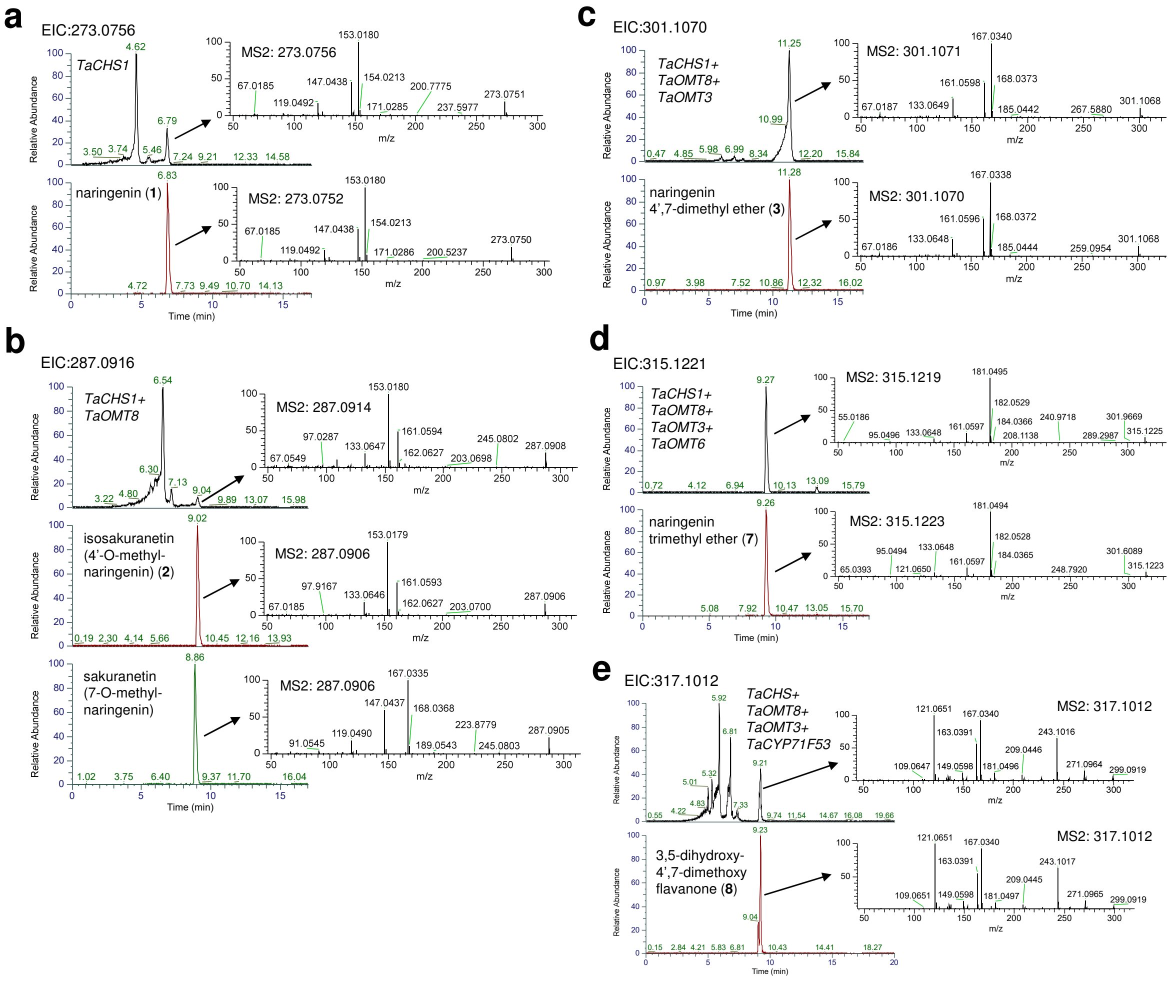

Supplementary Fig. 7. Extracted ion chromatograms and MS2 spectra of BGC4(5D) pathway intermediates in *Nicotiana benthamiana*. Chromatograms and MS2 spectra are shown for extracts of *N. benthamiana* leaves infiltrated with agrobacteria strains carrying expression vectors for genes from wheat BGC4(5D), and the corresponding commercial standards (panels A-D) or purified compound (panel E). **a**, expression of *TaCHS1* produces naringenin (**1**). **b**, co-infiltration of *TaCHS1* and *TaOMT8* produces isosakuranetin (**2**). **c**, co-infiltration of *TaCHS1*, *TaOMT8* and *TaOMT3* produces naringenin 4',7-dimethyl ether (**3**). **d**, co-infiltration of *TaCHS1*, *TaOMT8*, *TaOMT3* and *TaOMT6* produces naringenin trimethyl ether (**7**). **e**, co-infiltration of *TaCHS1*, *TaOMT8*, *TaOMT3* and *TaCYP71F53* produces 3,5-dihydroxy-4',7-dimethoxyflavanone (**8**).

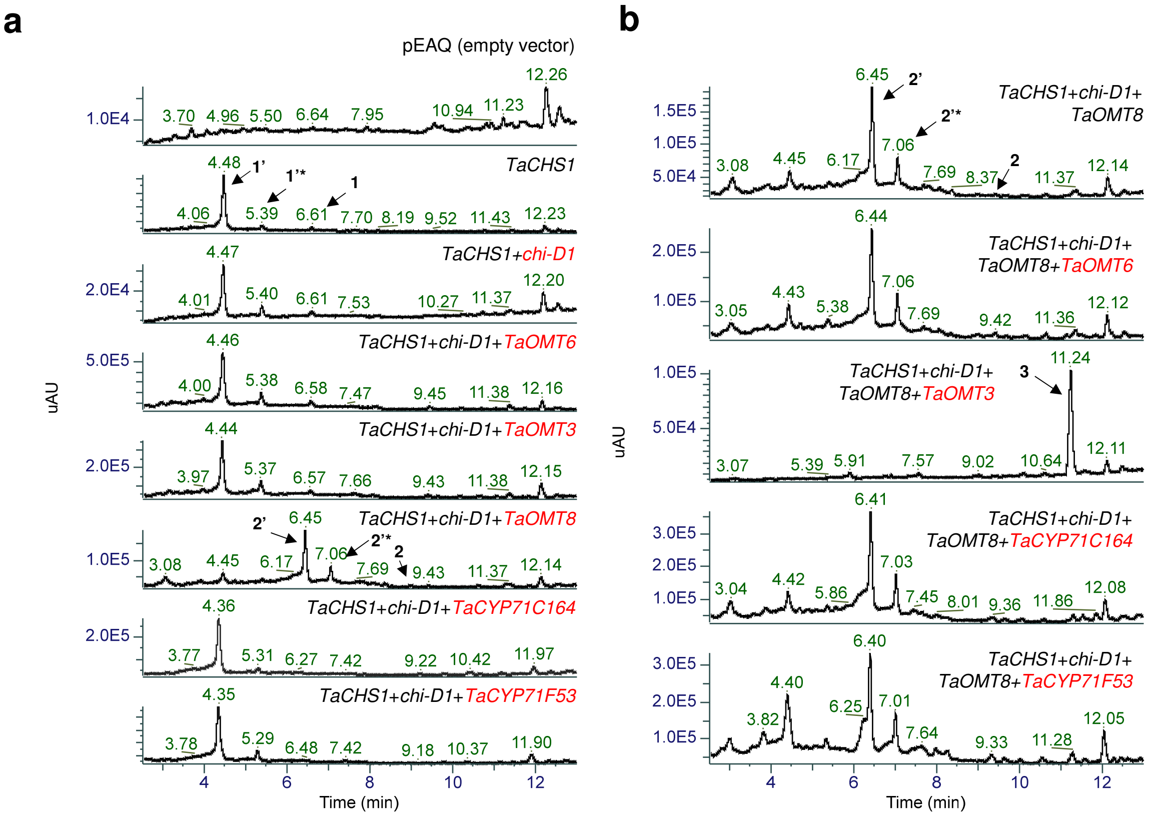

Supplementary Fig. 8. Total scan photo diode array (PDA) chromatograms of combinatorial transient expression experiments in *Nicotiana benthamiana*. **a**, expression of *TaCHS1* produces naringenin (**1**) and derivatives. Co-infiltration with *TaOMT8* produces isosakuranetin (**2**) and derivatives. **b**, additional expression of *TaOMT3* produces naringenin 4',7-dimethyl ether (**3**). Glycoside ('); diglycoside (''); malonyl-glycoside ('*). See Supplementary Table S1 for information on corresponding MS data of annotated peaks.

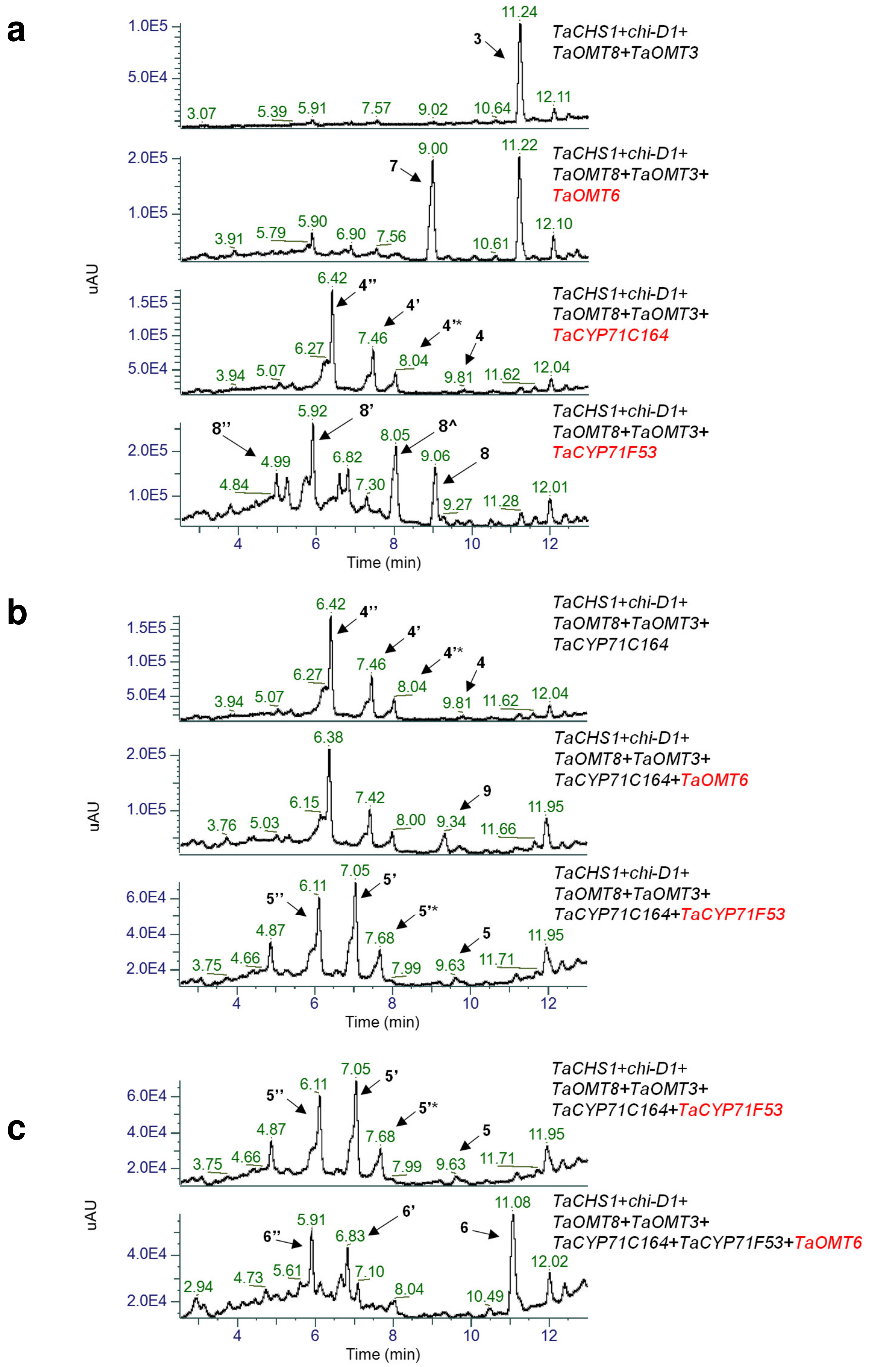

Supplementary Fig. 9. Total scan photo diode array (PDA) chromatograms of combinatorial transient expression experiments in *Nicotiana benthamiana*. **a**, additional expression of *TaOMT6*, *TaCYP71C164*, or *TaCYP71F53*, respectively converts naringenin 4',7-dimethyl ether (**3**) to naringenin trimethyl ether (**7**), artocarpanone A (**4**), or 3,5-dihydroxy-4',7-dimethoxyflavanone (**8**). **b**, additional expression of *TaOMT6* or *TaCYP71F53* respectively converts artocarpanone A (**4**) to 2',4',5-7-tetramethoxyflavanone (**9**), or 2'-*O*-demethyl-triticein (**5**). **c**, additional expression of *TaOMT6* converts 2'-*O*-demethyl-triticein (**5**) to triticein (**6**). Glycoside ('); diglycoside (''); malonyl-glycoside ('*); methyl ether (^). See Supplementary Table S1 for information on corresponding MS data of annotated peaks.

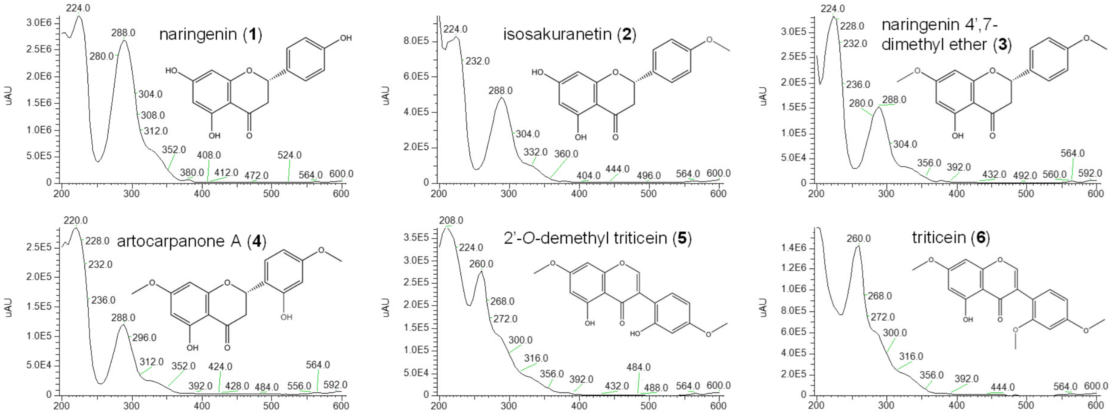

Supplementary Fig. 10. UV-VIS spectra of triticein pathway intermediates. Spectra are shown for three commercial standards (top row) and three purified compounds (bottom row).

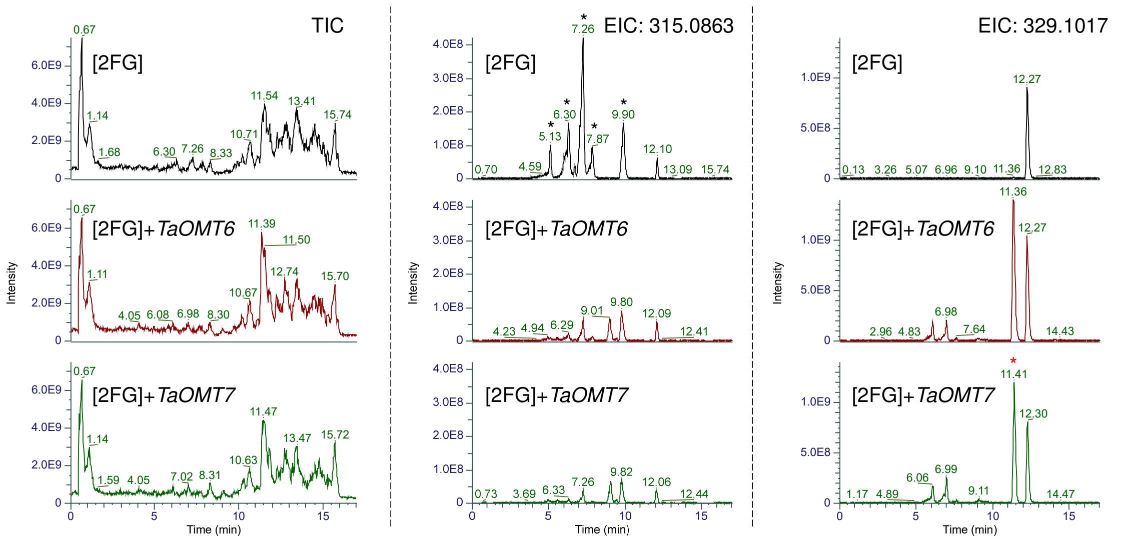

Supplementary Fig. 11. Comparison of TaOMT6 and TaOMT7 2'-*O*-methyltransferase activity on 2'-*O*-demethyl-triticein. LC-MS chromatograms of transient expression in *Nicotiana benthamiana*. Left- total ion chromatograms (TIC). Middle, extracted ion chromatograms for 2'-*O*-demethyl-triticein (**5**) (*m/z* 315.0863). 2'-*O*-demethyl-triticein (Rt 9.9) and its glycosylated forms are marked with black asterisks. Right- extracted ion chromatograms for triticein (*m/z* 329.1017), marked with red asterisk. 2FG, 2'-*O*-demethyl triticein-forming-genes.

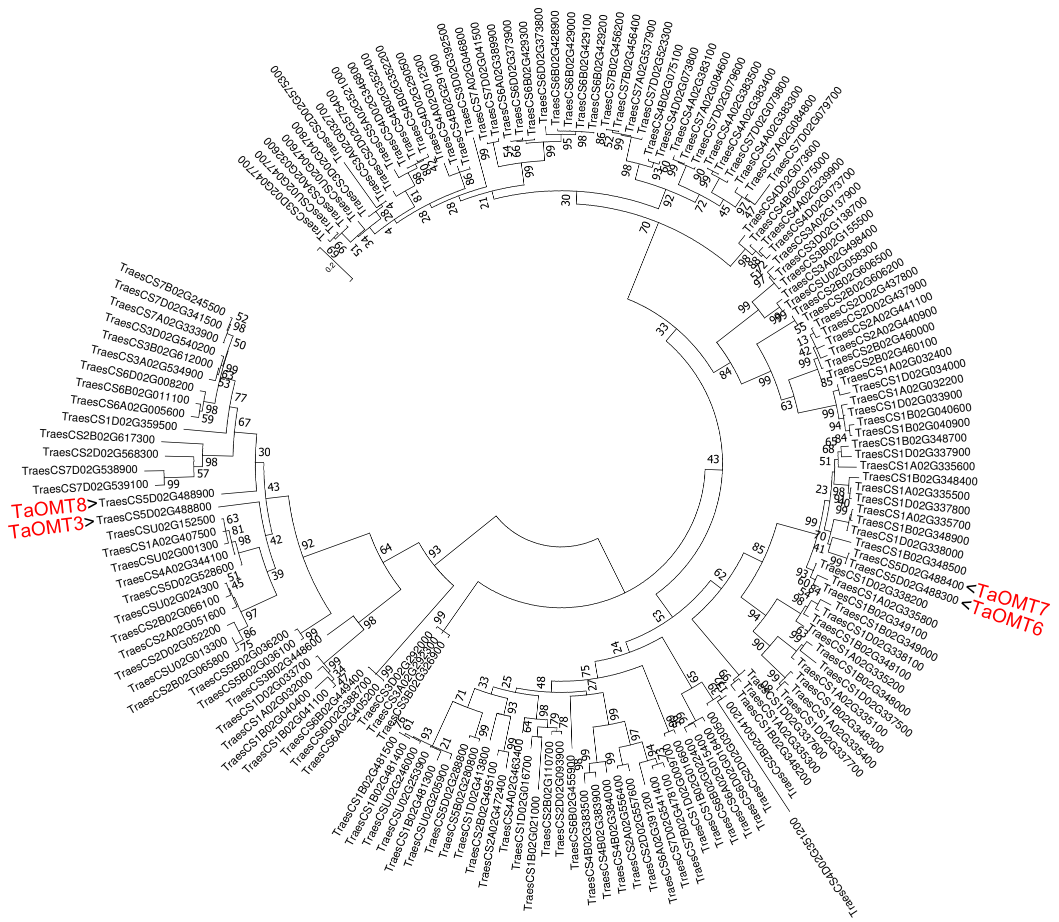

Supplementary Fig. 12. Phylogeny of *Triticum aestivum O*-methyltransferases. Maximum-likelihood tree was constructed for 165 *O*-methyltransferase protein sequences that include a 'Methyltransf_2' Pfam domain (PF00891). The Sequences were aligned with Muscle algorithm and the tree was constructed with MEGA7[9], using default parameters (with partial deletion, 95%) and 100 bootstraps.

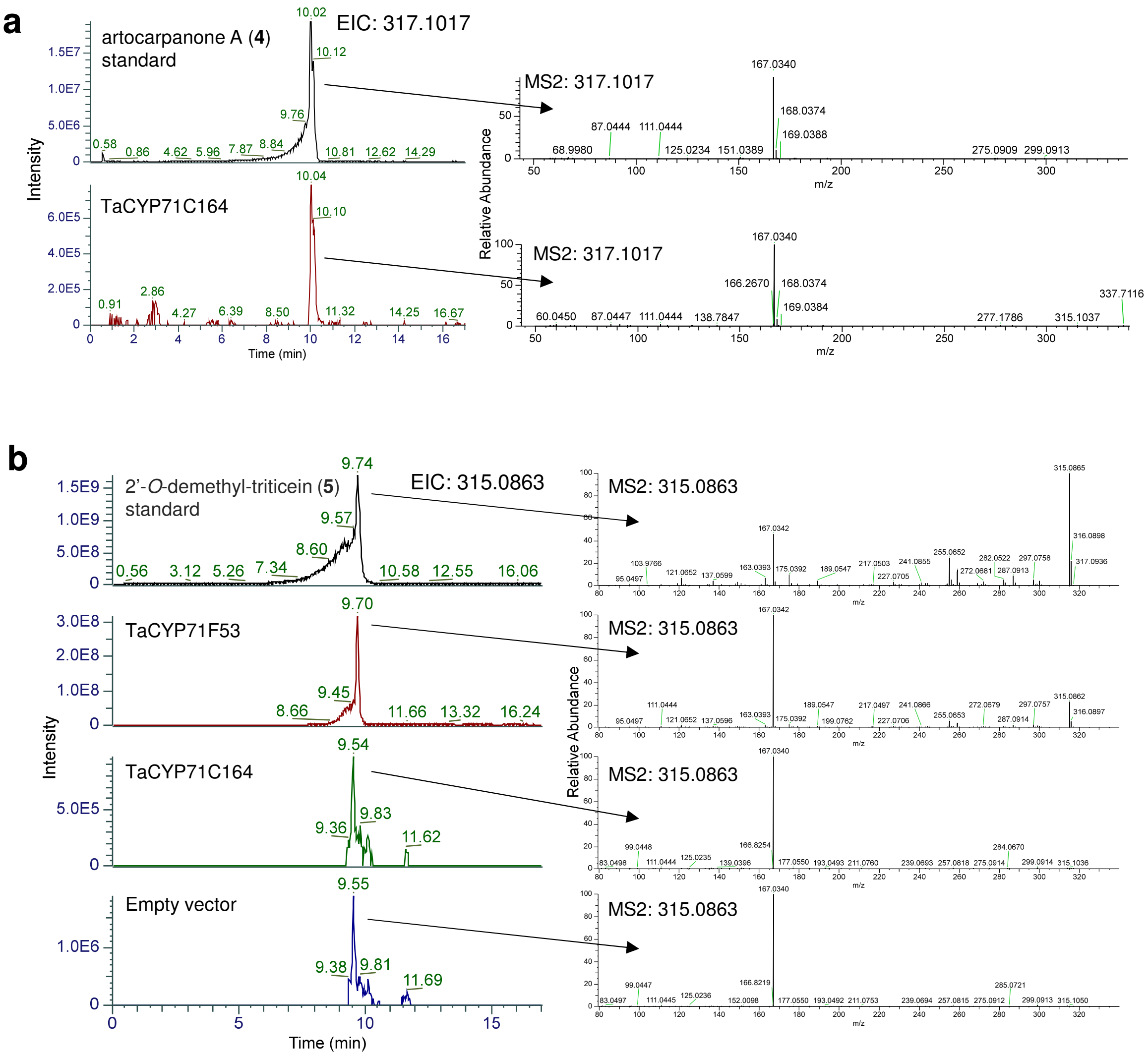

Supplementary Fig. 13. *TaCYP71C164* and *TaCYP71F53 in vitro* enzymatic assays. **a**, extracted ion chromatograms and MS2 spectra of the $[M+H]^+$ ion (*m/z*=317.1017) for 2',5-dihydroxy-4',7-dimethoxyflavanone (artocarpanone A) (**4**). Top image, purified artocarpanone A. Bottom image, reaction containing microsomal fractions of yeast expressing *TaCYP71C164*, and the substrate naringenin 4',7-dimethyl ether (**3**). **b**, extracted ion chromatograms and MS2 spectra of the $[M+H]^+$ ion (*m/z*=315.0863) for 2',5-dihydroxy-4',7-dimethoxyisoflavone (2'-*O*-demethyl-triticein) (**5**). Top image, purified 2'-*O*-demethyl-triticein. Bottom images, reactions containing microsomal fractions of yeast expressing *TaCYP71F53*, *TaCYP71C164*, or empty vector control, and the substrate artocarpanone A (**4**).

Cad0227 (TaCYP71F53)

MEG*LTLCFIAVSTLVAVWFSGGKSKPKKHLPPGPWTLPVIGSLHHVLSVLPHRTITELCRRHGPLMLLKLGEVP
TVVVSSAEAVEQVMKTNDIAFSYRRTTELQDIVGFGGKGIIFAPYDNRWRQMRKVCIMELLNSKQVKRMEDIRAE
EVGRLLRSIITATAVGATVNISQKAAALSNAVVTRAVFGGKFARQEEYLREINQLLELLGGFCLVDLFPSSRLVR
WFSTCERRTKKSCDLIQHIITGVLDERKVVRAAGDGACSTDDEDLLDVLLRLQEEDSLAYPLTTENITTVLFDIF
AAGTDTTGTALEWAMSELICHPEAMAKAQLEVREVLGHGRAIIGNSDLAKLHYLRMVIKEVLRLHPPGALLPRKT
REDCKIMGYDMLKDTNIYINVFAISRDPRYWNNPEEFNPGRFENNNVDYNGNSFEFTPFGGGRRQCPGIAFATSL
LEITLANFLYHFNWMLPGEASSASLDMSEKFGFTIGRTSNLHLKAIPYVRSTI

GGT TG**G** ---> GGT TG**A**
G W G *

Cad1793 (TaCYP71F53)

**MEGWLTLCFIAVSTLVAVWFSGGKSKPKKHLPPGPWTLPVIGSLHHVLSVLPHRTITELCRRHGPLMLLKLGEVP
TVVVSSAEAVEQVMKTNDIAFSYRRTTELQDIVGFGGKGIIFAPYDNRWRQMRKVCIMELLNSKQVKRMEDIRAE
EVGRLLRSIITATAVGATVNISQKAAALSNAVVTRAVFGGKFARQEEYLREINQLLELLGGFCLVDLFPSSRLVR
WFSTCERRTKKSCDLIQHIITGVLDERKVVRAAGDGACSTDDEDLLDVLLRLQEEDSLAYPLTTENITTVLFDIF
AAGTDTTGTALEWAMSELICHPEAMAKAQLEVREVLGHG***AIIGNSDLAKLHYLRMVIKEVLRLHPPGALLPRKT
REDCKIMGYDMLKDTNIYINVFAISRDPRYWNNPEEFNPGRFENNNVDYNGNSFEFTPFGGGRRQCPGIAFATSL
LEITLANFLYHFNWMLPGEASSASLDMSEKFGFTIGRTSNLHLKAIPYVRSTI

GGC **C**GA ---> GGC **T**GA
G R G *

Cad1682 (TaOMT3)

MGSTAVEKVAVATGDEEACMYAVKLAAASILPMTLKNAIELGMLEILVGAGGKMLSPSEVAA*LPSKANPEAPVM
VDRMLRLLASNNVVSCEVEEGKDGLLARRYGPAPVCKWLTPNEDGASMAGLLLMTHDKVTMESWYYLKDVALEGG
QPFHRAHGMTAYEYNSTDPRANCLFNEAMLNHSTIITKKLLEFYRGFDNVETLVDVAGGVGATAHAITSKYPHIK
GVNFDLPHVISEAPPYPGVQHIAGDMFKKVPSGDAILLKWILHNWTDDYCMTLLRNCYDALPMNGKVVIVEGILP
VKPDAMPSTQTMFQVDMMMLLHTAGGKERELSEFEELAKGAGFSTVKTSYIYSTAWVIEFVK

GCG **C**AG ---> GCG **T**AG
A Q A *

Cad1684 (TaOMT8)

**MGSISDDEAACIYAMQLAAAAVLPMTLKNAIELGMIEILVGAGGKMLSPSEVAAQLPSTANAEAPAIVDRMLRLL
ASHNVVSCEVEEGKDGRLARRYGTAPVCKWLSPDEDGVSLAPMVLLNDKVMLES***YHLKDTVLNGGLPFEKAYGM
TAFDYQGTDPRFNRVFNEAMKTHSMIITKKLLEFYKGFDGVGTLVDVGGGVGATIHTILCRYPSIEGVNFDLPHV
ISEAPSFAGVHHIGGDMFKKVPSGDAILMKWILHDWNDEHCTTLLSNCYDALPAHGKVVIVEYILPVKPDETATA
QRSFEADMIMLTHTPGGKERYLREYEELARSAGFASVKATYVYSNIWVIELNK

AGC TG**G** ---> AGC TG**A**
S W S *

Supplementary Fig. 14. DNA sequence mutations in four independent cv. 'Cadenza' wheat TILLING lines, resulting in premature stop codons for three genes from BGC4(5D).

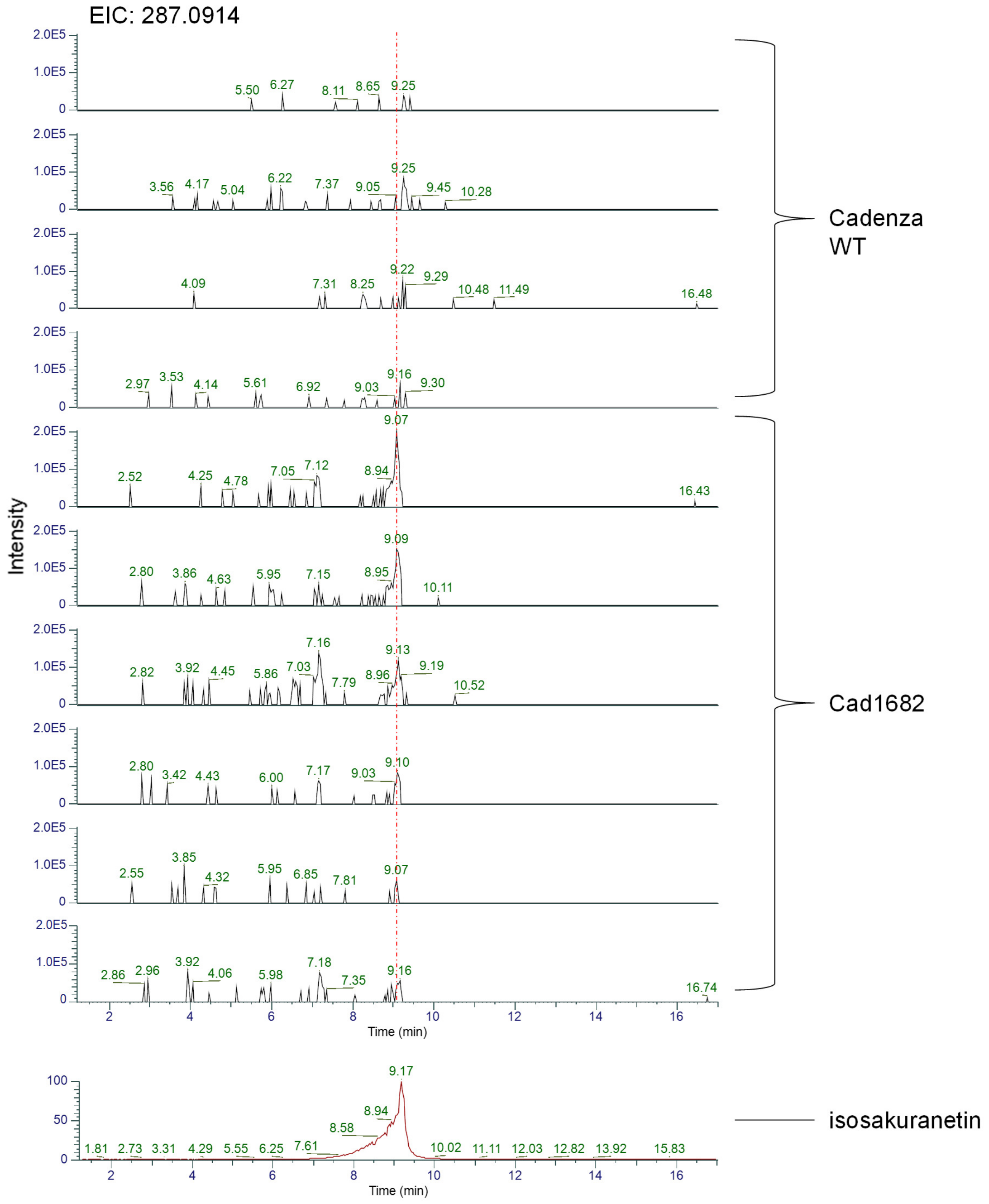

Supplementary Fig. 15. Extracted ion chromatograms of the [M+H]$^+$ ion for isosakuranetin. EIC are shown for blade extracts from four plants of the Cadenza parent line, and six plants of the Cad1682 line. Y-axes are linked. EIC of isosakuranetin (**2**) standard is shown for reference.

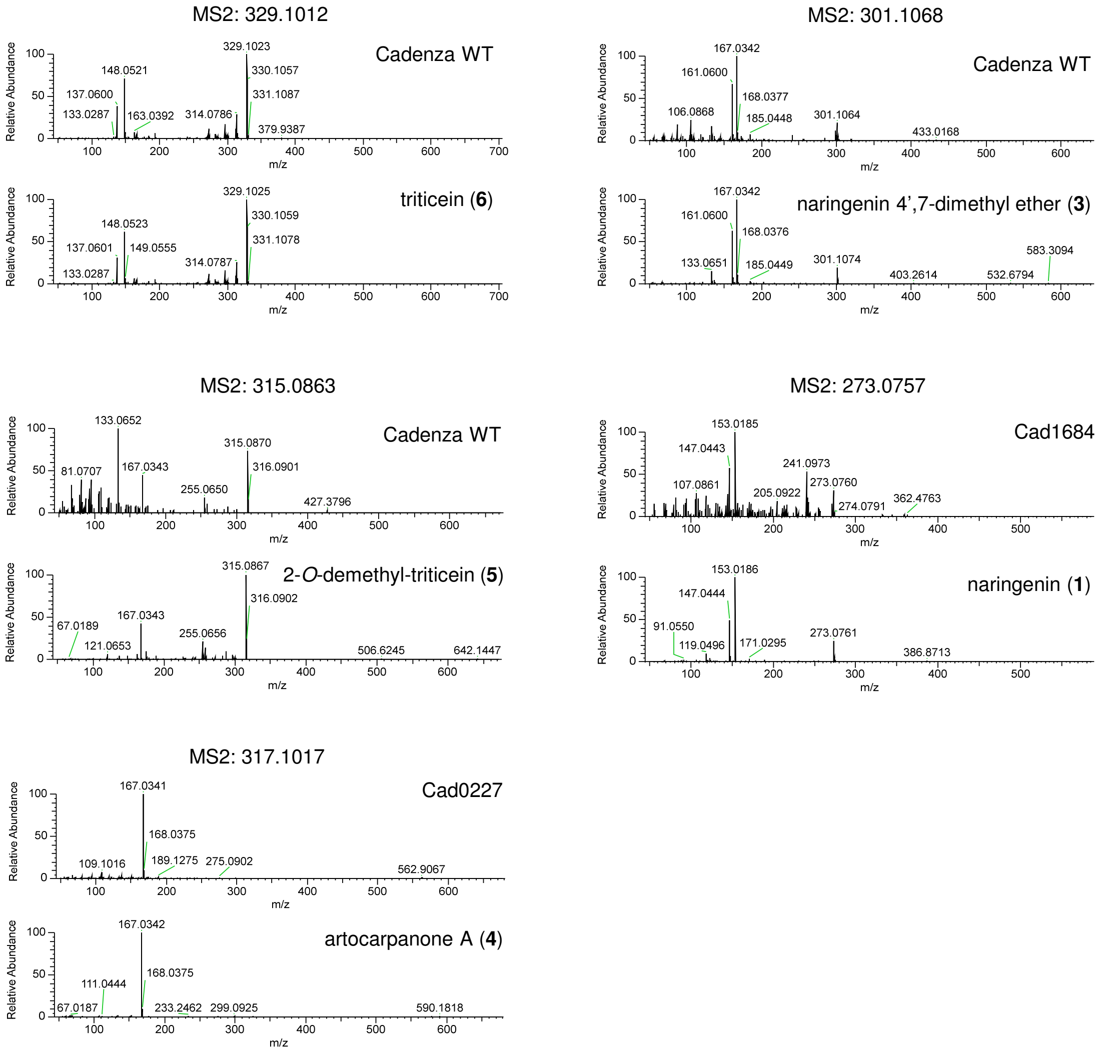

Supplementary Fig. 16. Selected MS2 spectra from wheat TILLING lines blade extracts. Triticein (**6**) and pathway intermediates were identified in extracts from wheat Cadenza wildtype parent or TILLING mutant lines, based on MS2 fragmentation of ions with corresponding accurate masses and retention times, and comparison with commercial standards or purified compounds.

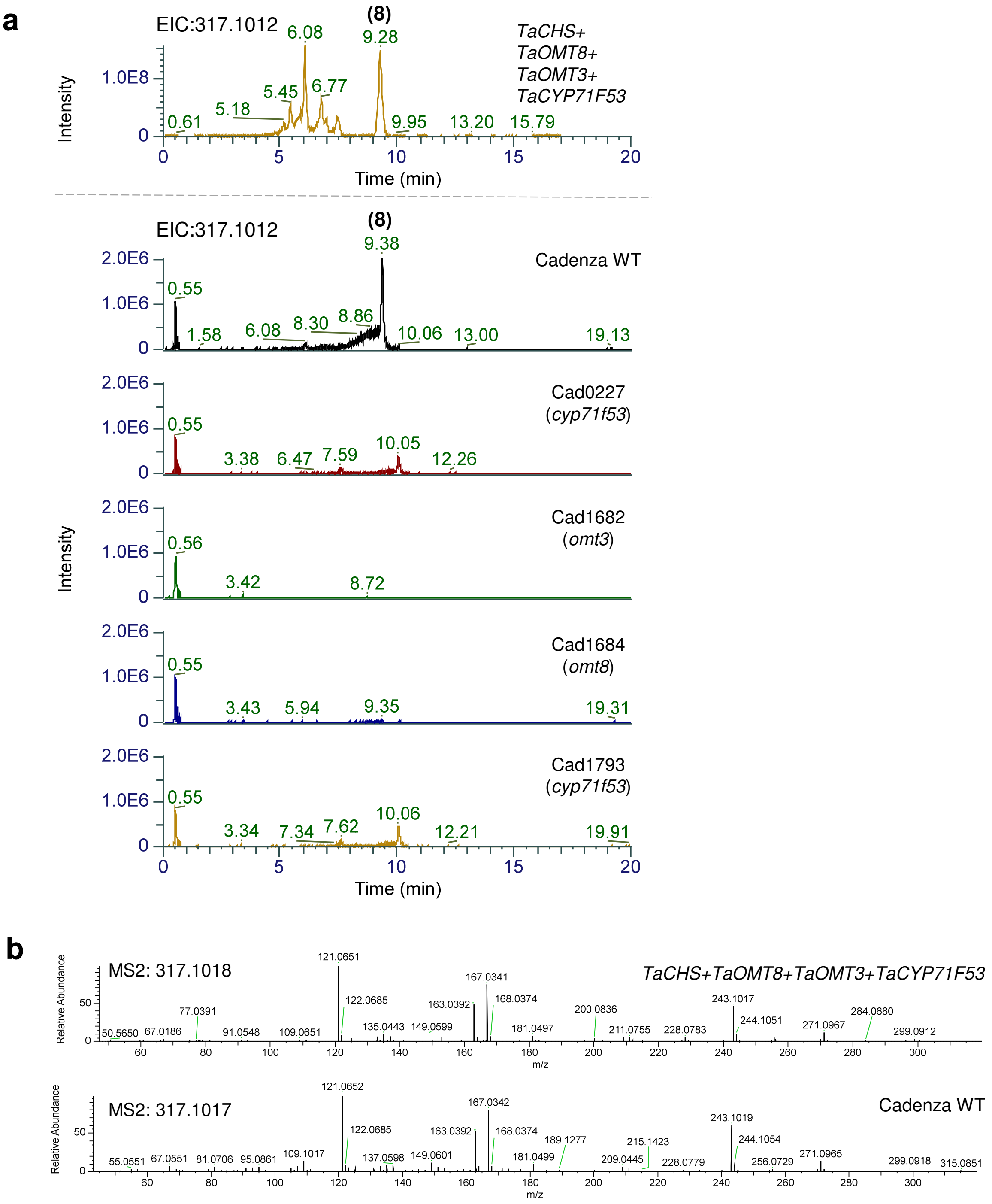

Supplementary Fig. 17. 3,5-dihydroxy-4',7-dimethoxyflavanone (8) is detected in Cadenza wildtype lines. a, extracted ion chromatograms of the **[M+H]$^+$** ion of (**8**), in agroinfiltrated *N. benthamiana* leaves (top chromatogram), and wheat Cadenza TILLING lines (bottom five chromatograms). **b,** MS2 spectra for corresponding peaks in *N. benthamiana* (top) and wheat (bottom).

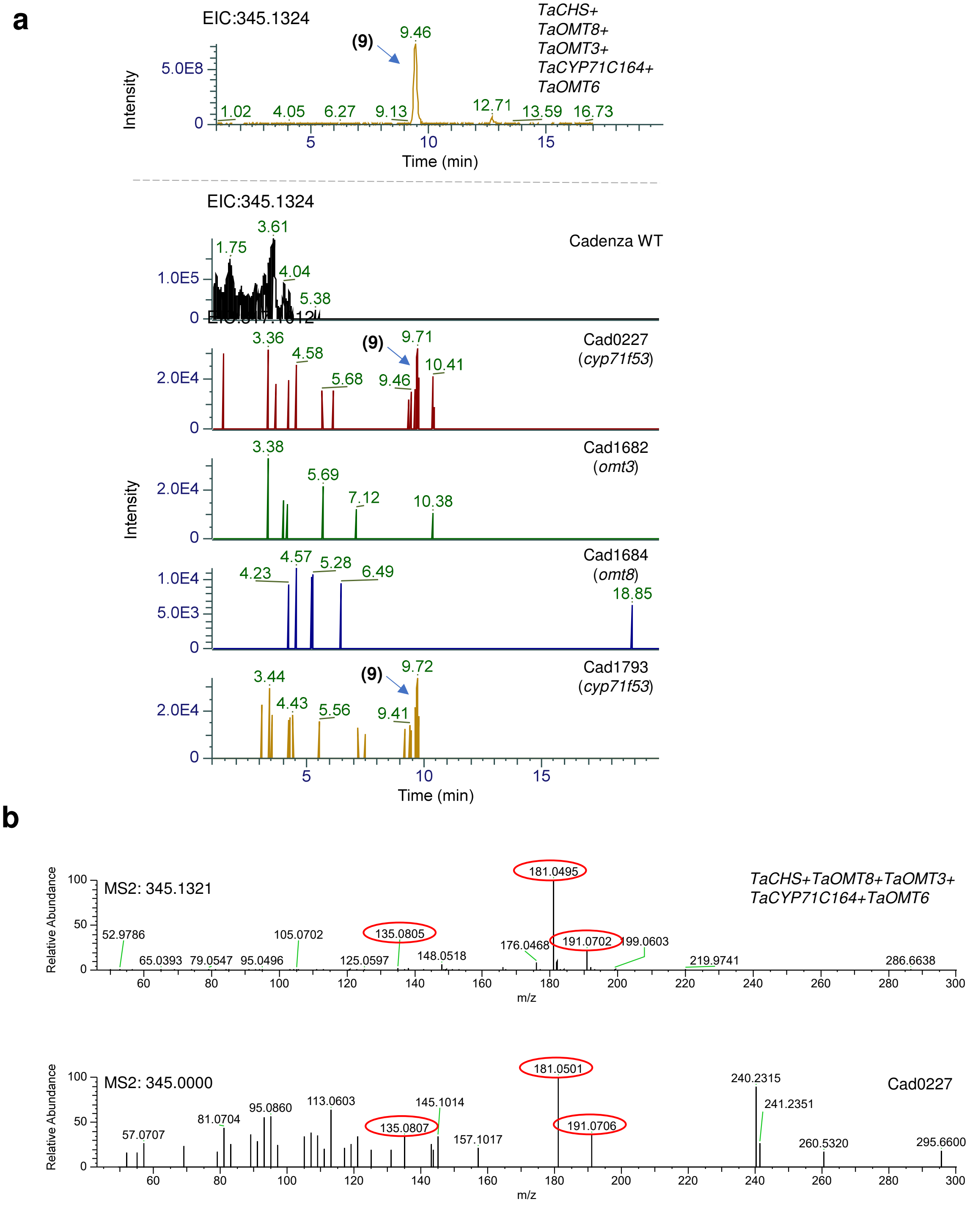

Supplementary Fig. 18. 2',4',5,7-tetramethoxyflavanone (9) is detected in Cadenza *cyp71f53* mutant lines. a, extracted ion chromatograms of the **[M+H]⁺** ion of (**9**), in agroinfiltrated *N. benthamiana* leaves (top chromatogram), and wheat Cadenza TILLING lines (bottom five chromatograms). **b,** MS2 spectra for corresponding peaks in *N. benthamiana* (top) and wheat (bottom).

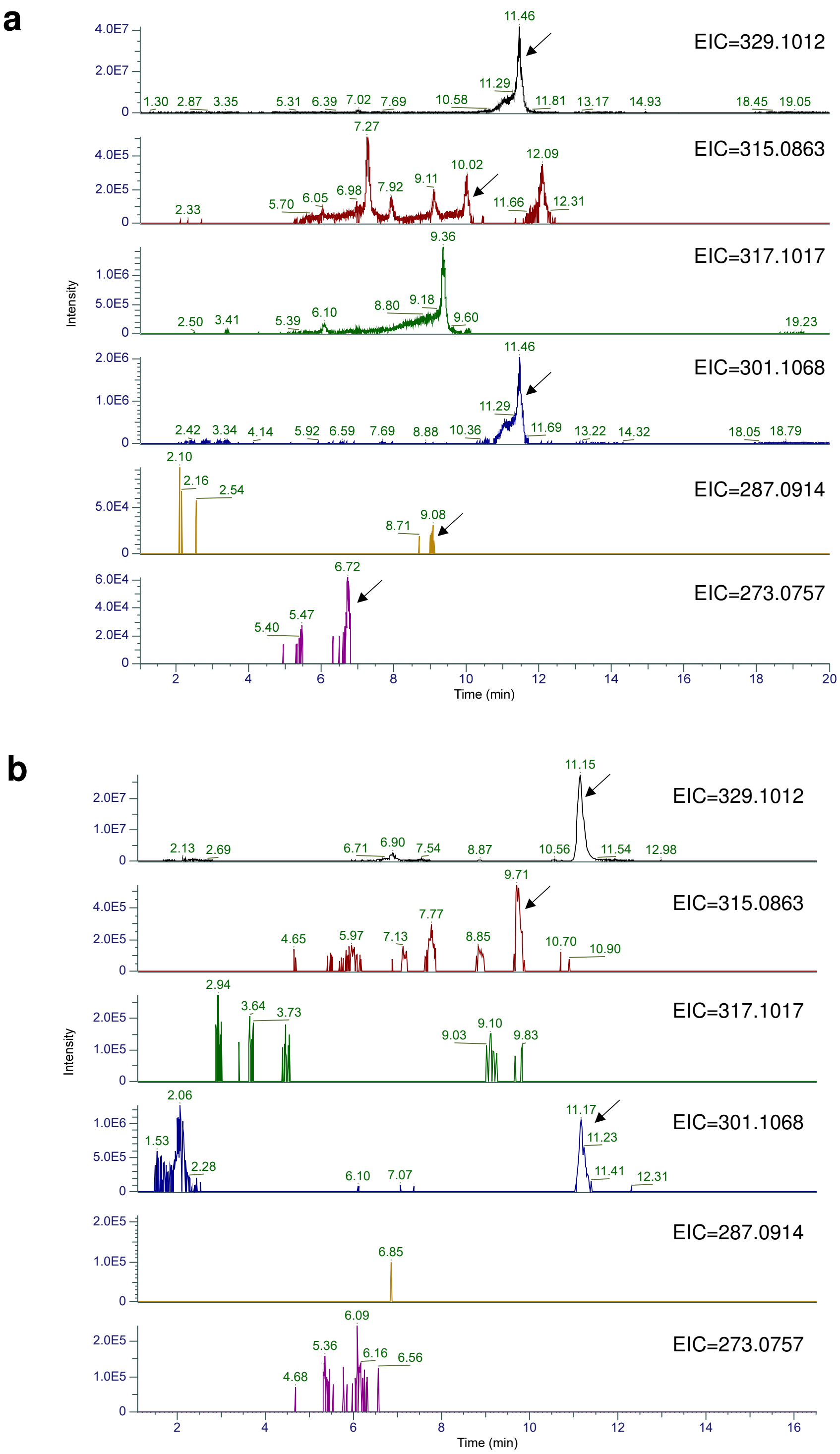

Supplementary Fig. 19. Triticein is more abundant than pathway intermediates in blades from both Chinese Spring and Cadenza cultivars. From top to bottom, extracted ion chromatograms of masses corresponding with $[M+H]^+$ ions for triticein (**6**), 2'-*O*-demethyl-triticein (**5**), artocarpanone A (**4**), naringenin 4',7-dimethyl ether (**3**), isosakuranetin (**2**), naringenin (**1**). Arrows mark peaks representing the relevant compounds, where observed. **a**, Cadenza. **b**, Chinese Spring.

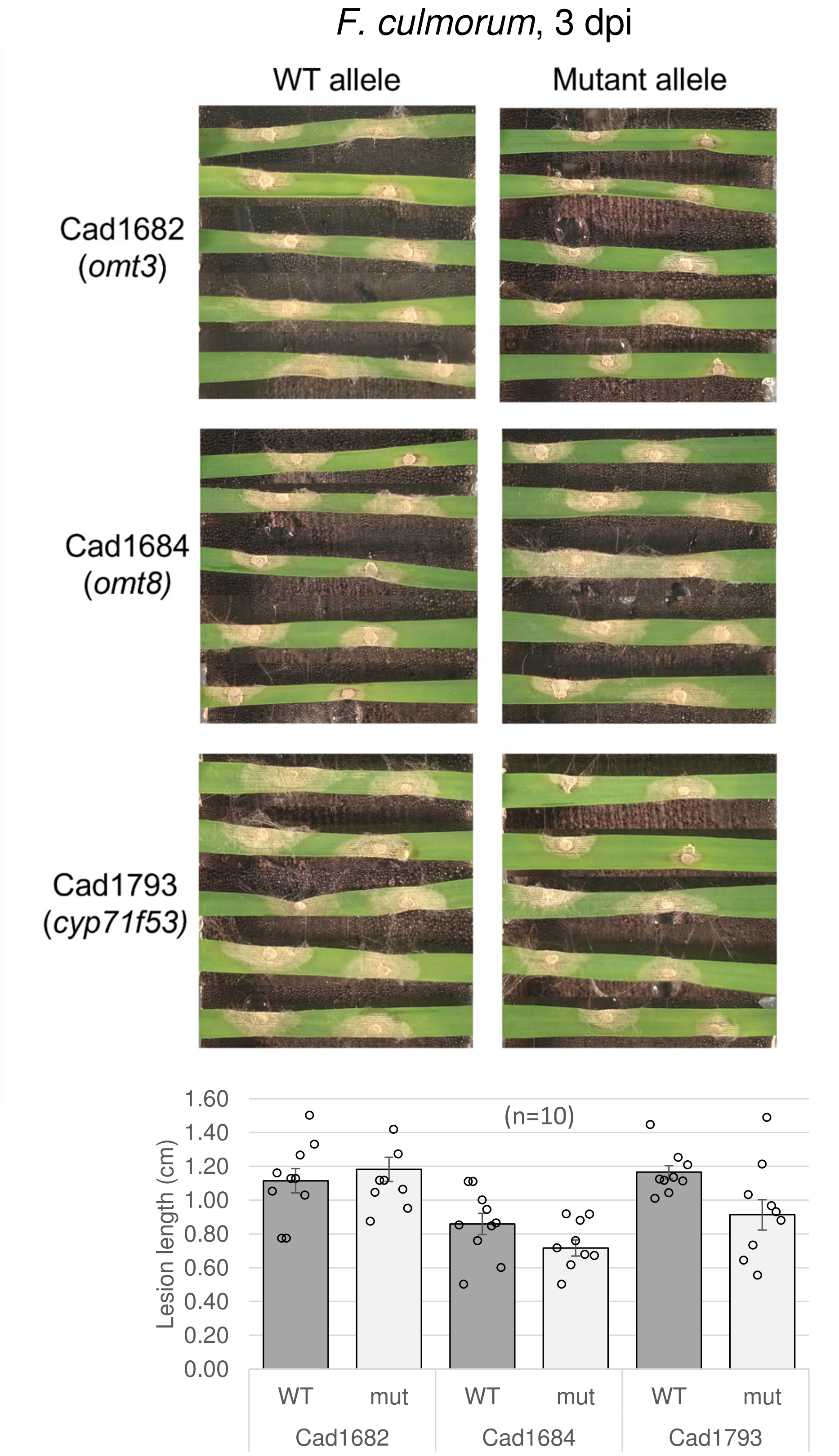

Supplementary Fig. 20. *Fusarium culmorum* infection of wheat blades. Detached leaves from wheat TILLING lines (cv. Cadenza) were infected with *F. culmorum* (isolate FC2021). Lesion lengths across infection points were measured 3 days post infection. n=10 (number of lesions measured). Source data are provided as a Source Data file.

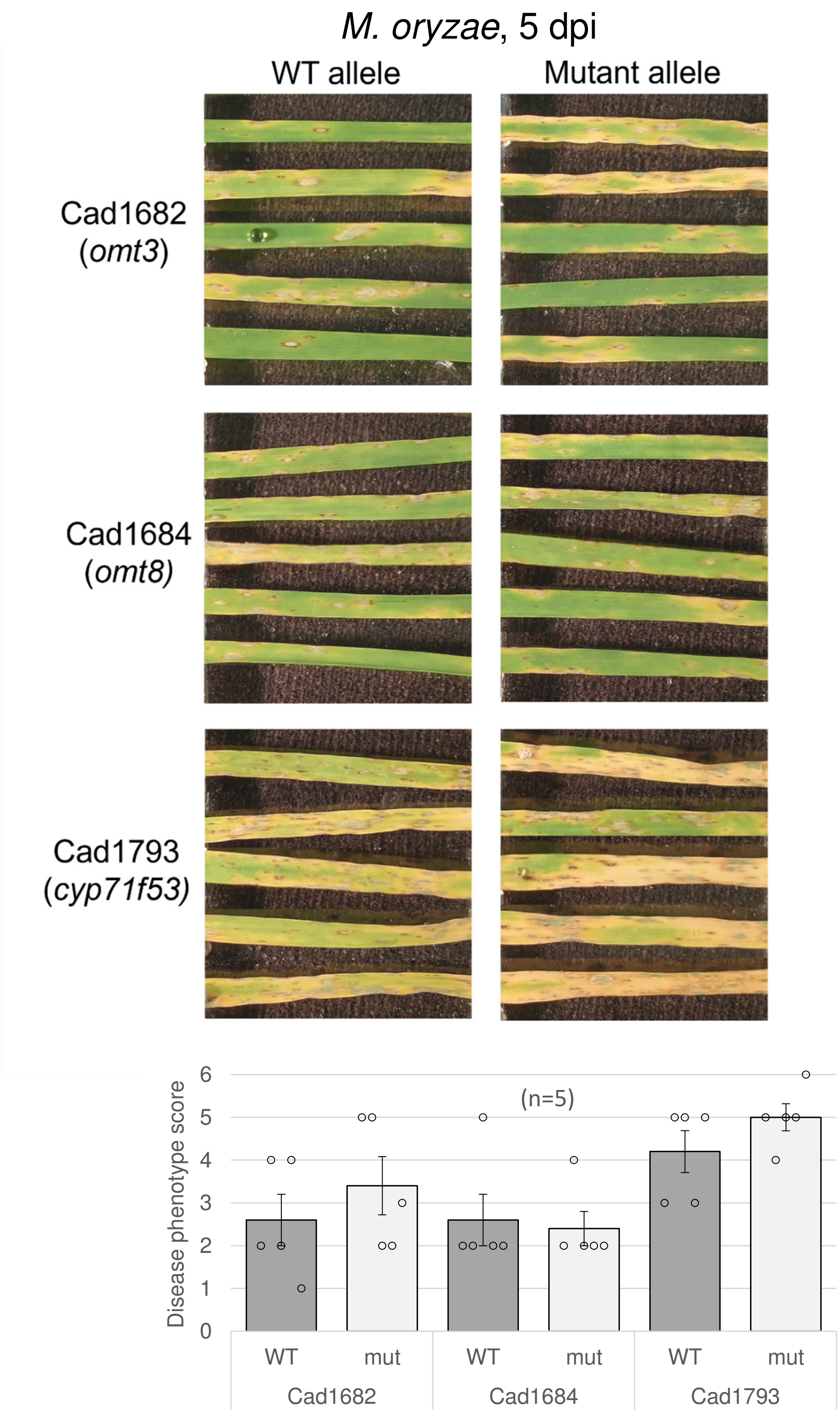

Supplementary Fig. 21. *Magnaporthe oryzae* infection of wheat blades. Detached leaves from wheat TILLING lines (cv. Cadenza) were infected with *M. oryzae* (isolate BTJ4P). Disease phenotype was scored five days post infection, based on a scale of 0 (lowest susceptibility) to 6 (highest susceptibility)[10]. n=5 (number of blades scored). Source data are provided as a Source Data file.

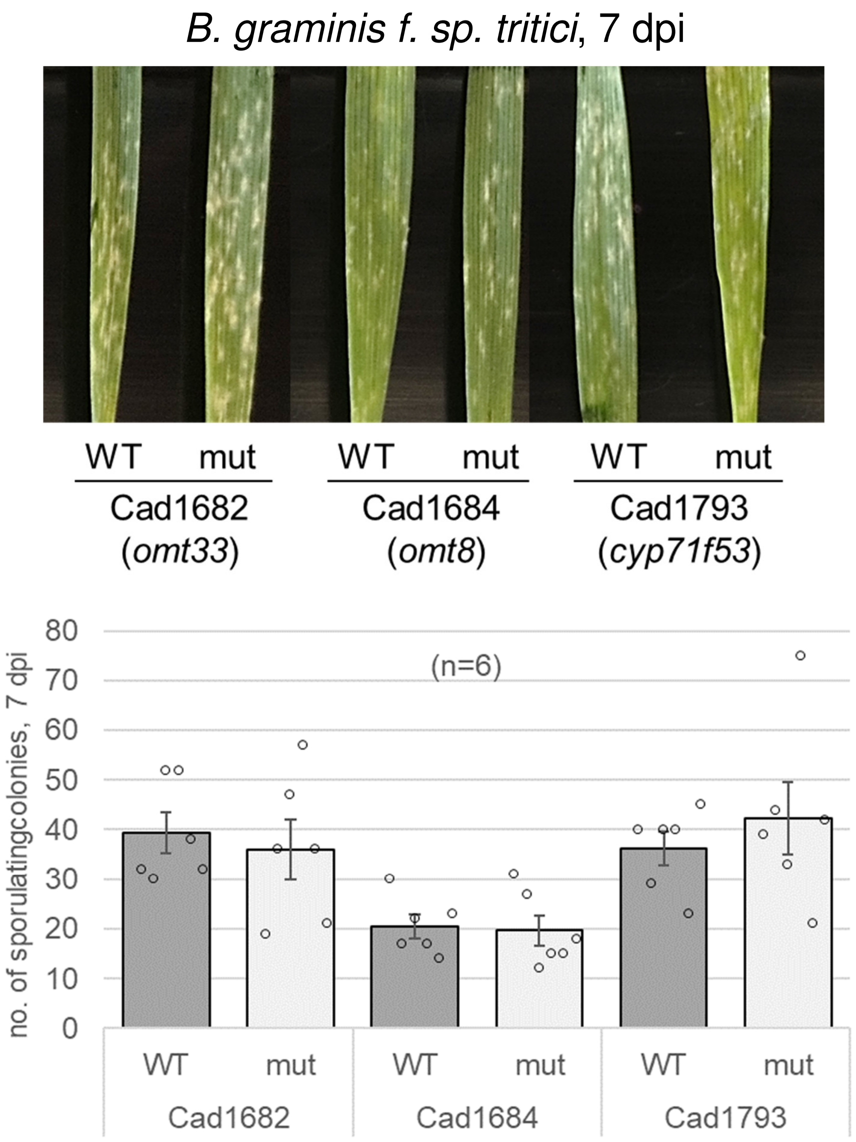

Supplementary Fig. 22. Powdery mildew infection of wheat blades. Detached leaves from wheat TILLING lines (cv. Cadenza) were infected with powdery mildew (*B. graminis f. sp. tritici*, isolate CAW14S6313). Observable sporulating colonies on the blade segments were counted 7 days post infection. n=6 (number of blades analyzed). Source data are provided as a Source Data file.

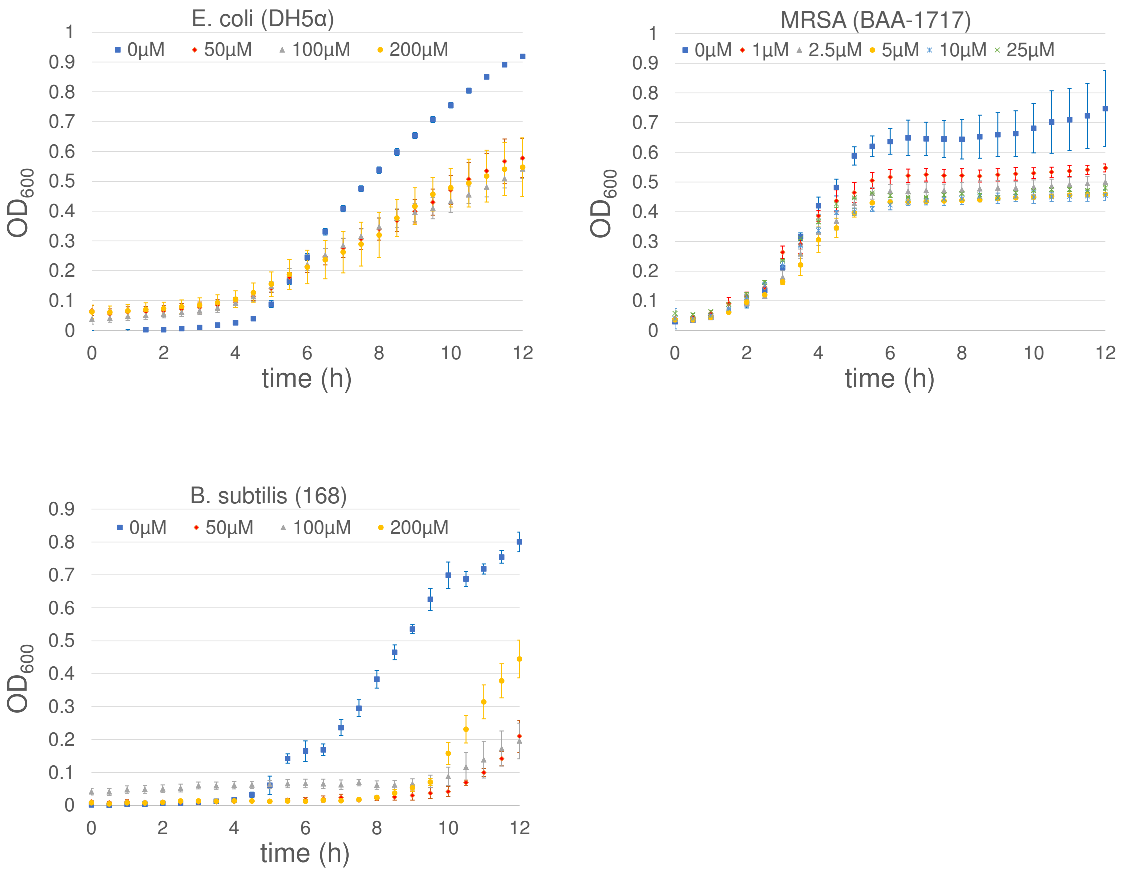

Supplementary Fig. 23. Bacterial growth inhibition assays in liquid LB media containing 50-200 μM triticein, or 1-25 μM triticein. n=3 (number of biological replicates). Source data are provided as a Source Data file.

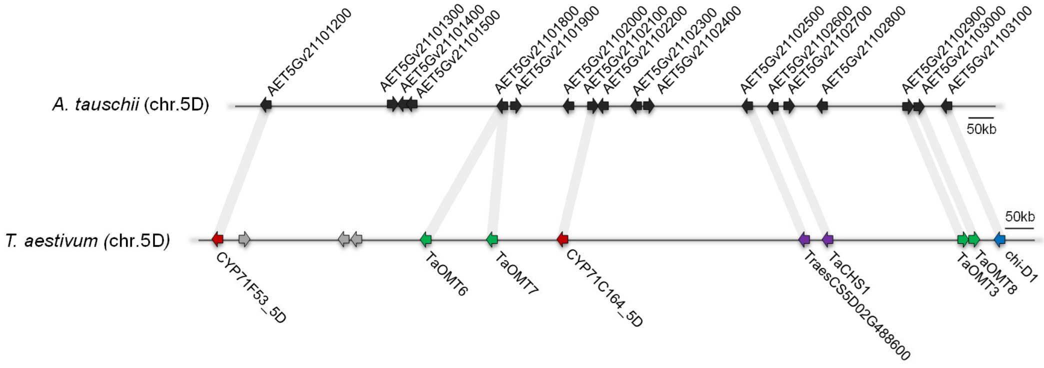

Supplementary Fig. 24. Wheat BGC4(5D) orthologous gene cluster on *Aegilops tauschii* chromosome 5D. Matching orthologs are marked with grey lines.

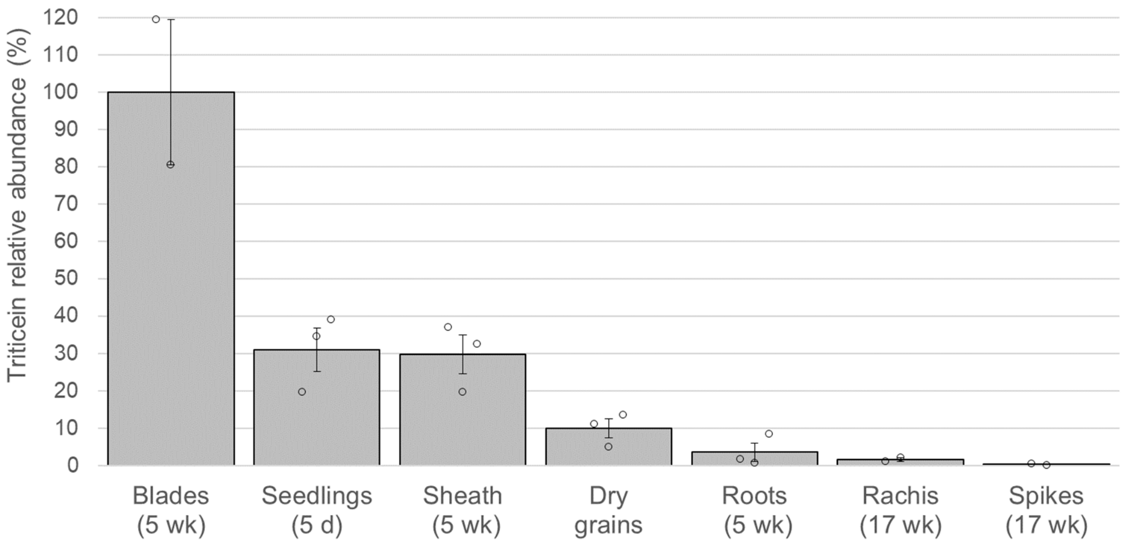

Supplementary Fig. 25. Relative abundance of triticein in tissues from MeJA-treated 'Chinese Spring' plants. Relative abundance is based on peak areas from LC-MS chromatograms, normalized by internal standard, digitoxin. All tissues, excluding dry grains, are from methyl jasmonate-treated plants. Age of plants sampled is shown in brackets. d, days; wk, weeks. n=2/n=3 (number of biological replicates). Source data are provided as a Source Data file.

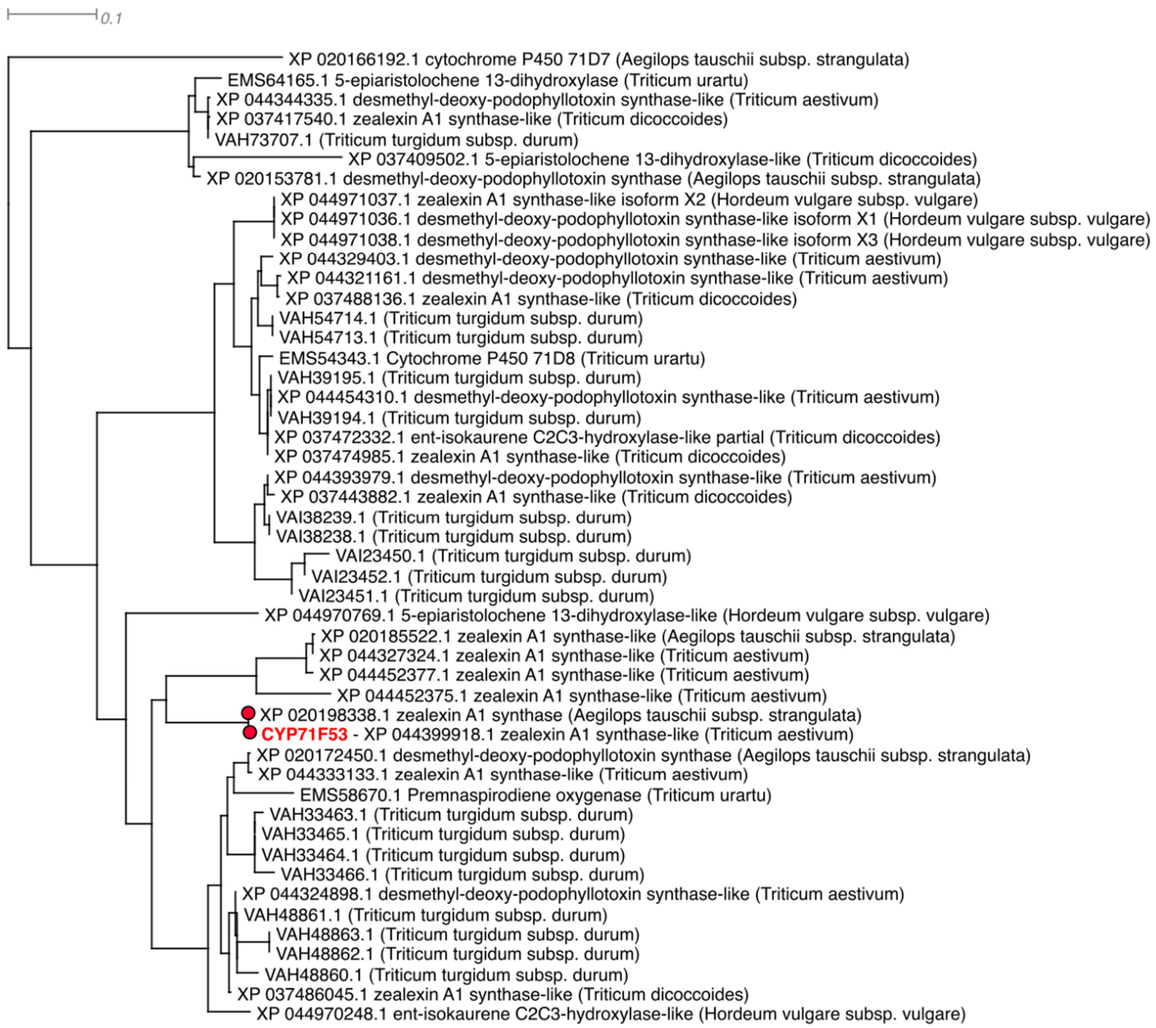

Supplementary Fig. 26. Extracted section from maximum likelihood phylogenetic tree of CYP71 sequences from selected grass genomes. Cytochrome P450 sequences were mined from selected grass genomes using HMMER and Pfam profile p450 (PF00067), and genomic data accessed at Ensembl Plants (https://plants.ensembl.org/index.html): *Aegilops tauschii subsp. strangulata, Brachypodium distachyon, Hordeum vulgare subsp. vulgare, Oryza sativa Indica, Oryza sativa japonica, Panicum virgatum, Setaria italica, Sorghum bicolor, Triticum aestivum, Triticum dicoccoides, Triticum turgidum subsp. durum, Triticum urartu, Zea mays*. Sequences were aligned with MAFTT[11], using automatic parameters and a draft phylogeny generated with FastTree 2[12], using standard parameters. The draft phylogeny was used to select putative CYP71 sequences for higher quality alignment and phylogenetic analysis. Selected CYP71 peptide sequences were next aligned with MAFTT[11], using the FFT-NS-i model with a maximum of 1000 iterations. The phylogenetic tree was generated from alignments with RaXML[13], using the PROTGAMMAAUTO model and 100 rapid bootstraps. *TaCYP71F53* and its ortholog in *Aegilops tauschii* are marked.

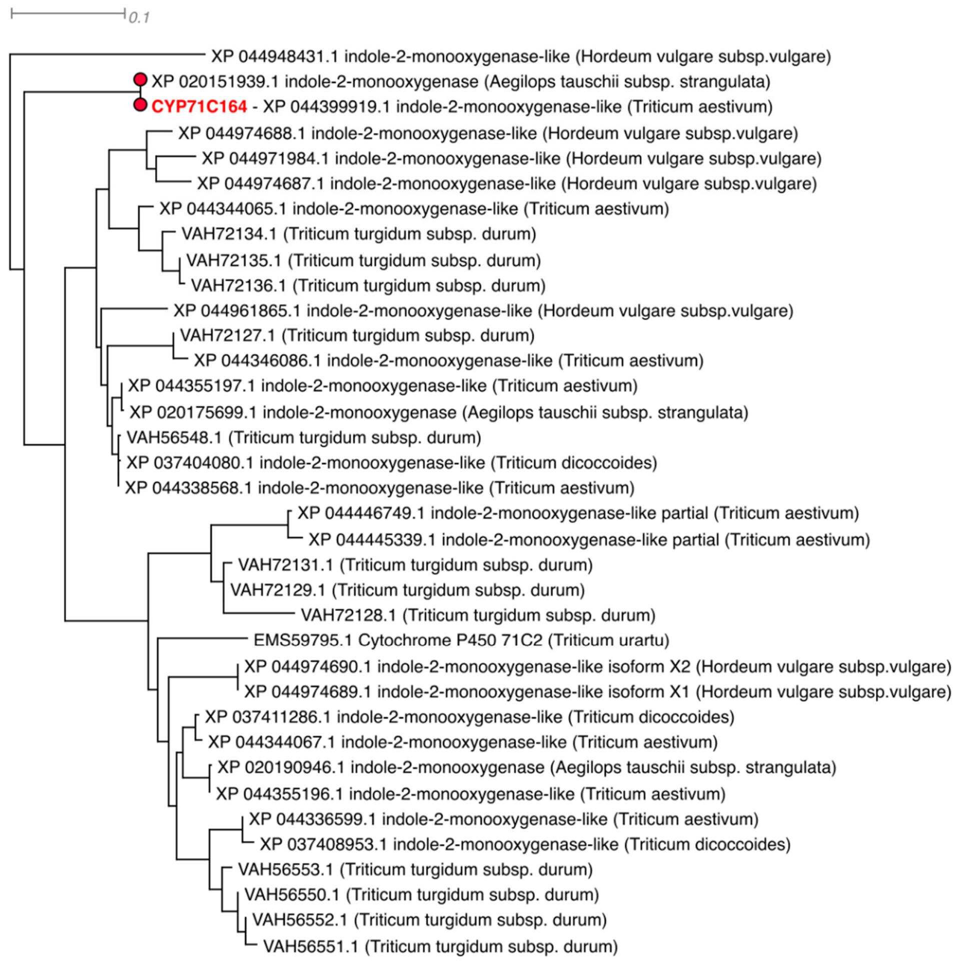

Supplementary Fig. 27. Extracted section from maximum likelihood phylogenetic tree of CYP71 sequences from selected grass genomes. See details described in Supplementary Fig. 21. *TaCYP71C164* and its ortholog in *Aegilops tauschii* are marked.

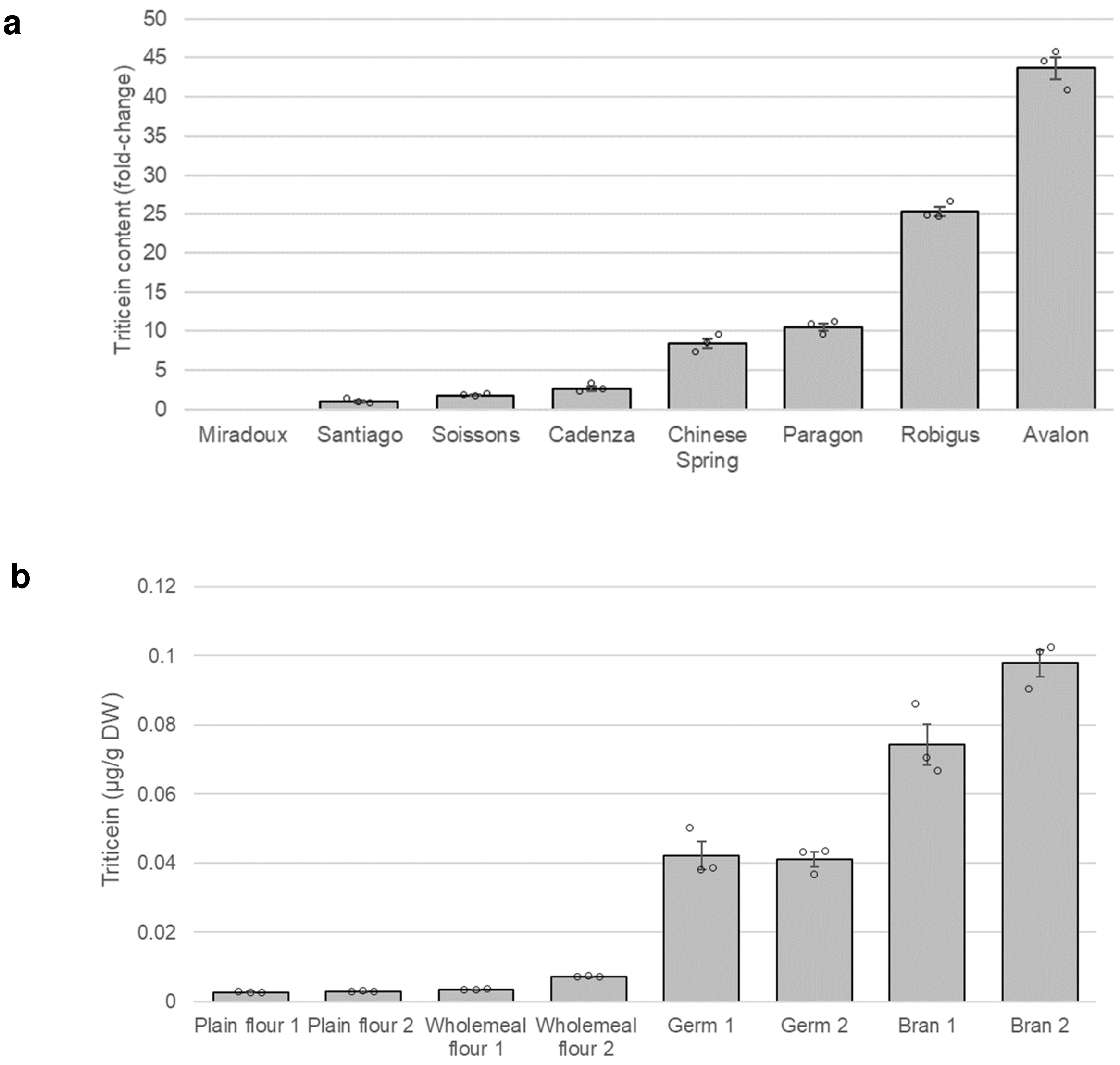

Supplementary Fig. 28. Triticein quantification in wheat grains and flours. a, relative quantification of triticein (**6**) content in grains from wheat landrace ‘Chinese Spring’, durum wheat cultivar ‘Miradoux’, and six commercial hexaploid wheat cultivars. Mean fold-change values compared to ‘Santiago’ ± SEM of three biological replicates are presented. **b**, quantification of triticein (**6**) content in commercial wheat flours. Mean values ± SEM of three technical replicates are presented. n=3 (biological replicates in panel **a**, technical replicates in panel **b**). Source data are provided as a Source Data file.

a

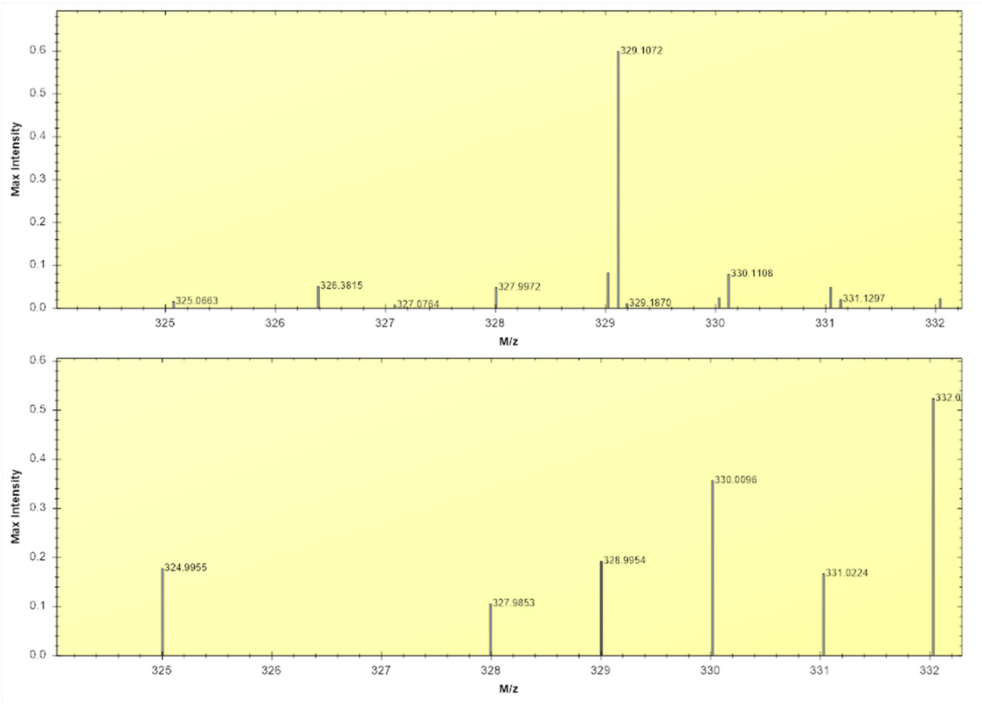

b

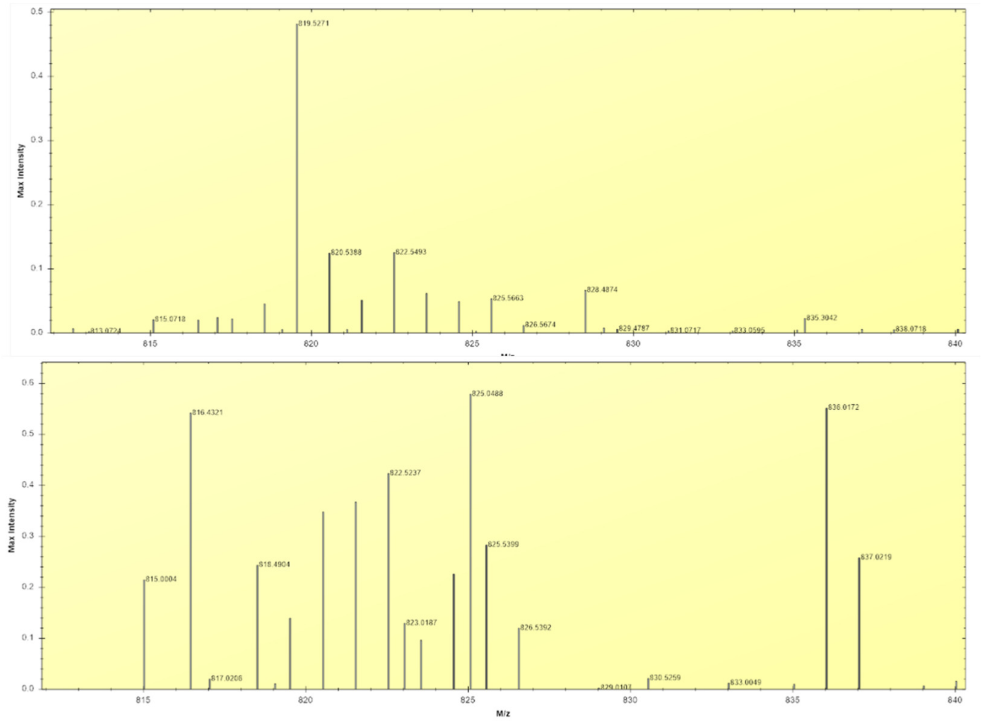

Supplementary Fig. 29. Extract of MALDI-MSI mass spectra. a, mass spectra at range of *m/z* 320 to 333. **b**, mass spectra at range of *m/z* 812 to 840. Images were generated from scans of cross-sectioned grains of *Triticum aestivum* (top image in each panel) and *Triticum turgidum subsp. durum* (bottom image in each panel). An $[M+H]^+$ ion for triticein (**6**) (*m/z* 329.1) is undetected in *T. turgidum* scans. The phosphatidylcholine ion (*m/z* 822.5) is present in both samples.

Gene ID	Annotation	Gene name in this study	Genomic location	Strand
**TraesCS5D02G487900**	**Cytochrome P450**	***TaCYP71F53***	**5D: 522,758,805-522,761,728**	**rev**
TraesCS5D02G488000	Pre-mRNA-splicing ATP-dependent RNA helicase PRP28		5D: 522,788,154-522,788,420	fwd
TraesCS5D02G488100	Neuroblastoma-amplified sequence protein		5D: 523,014,862-523,015,914	rev
TraesCS5D02G488200	50S ribosomal protein L32, chloroplastic		5D: 523,037,174-523,037,365	rev
**TraesCS5D02G488300**	**O-methyltransferase-like protein**	***TaOMT6***	**5D: 523,185,997-523,187,567**	**rev**
**TraesCS5D02G488400**	**O-methyltransferase-like protein**	***TaOMT7***	**5D: 523,313,281-523,314,724**	**rev**
**TraesCS5D02G488500**	**Cytochrome P450**	***TaCYP71C164***	**5D: 523,451,905-523,454,959**	**rev**
TraesCS5D02G488600	Chalcone synthase		5D: 523,938,040-523,940,039	rev
**TraesCS5D02G488700**	**Chalcone synthase***	***TaCHS1***	**5D: 523,991,850-523,993,876**	**rev**
**TraesCS5D02G488800**	**O-methyltransferase**	***TaOMT3***	**5D: 524,249,356-524,251,095**	**fwd**
**TraesCS5D02G488900**	**O-methyltransferase**	***TaOMT8***	**5D: 524,259,452-524,261,298**	**fwd**
TraesCS5D02G489000	Chalcone-flavanone isomerase family protein	*chi-D1*	5D: 524,334,560-524,335,791	rev

Table S1. Genes comprising BGC4(5D) in *Triticum aestivum* (cv. Chinese Spring). Gene IDs (IWGSC RefSeqv1.1) and genomic locations were extracted from EnsemblPlants (https://plants.ensembl.org/). In bold: genes that are co-expressed (r-val>0.7) with the *TaCHS1* bait gene (asterisked).

Compound	Compound number	Gene expression combination	Elemental composition [M+H]$^+$	Calculated mass [M+H]$^+$	Observed mass [M+H]$^+$	Delta [ppm]	Rt PDA (min)	λmax (nm)
**Naringenin**	**1**	TaCHS+chi-D1	C15H13O5	273.0757	273.0752	-2.14	6.61	288
naringenin (glycoside)	1'		C21H23O10	435.1286	435.1274	-2.60	4.48	284
naringenin (malonyl-glycoside)	1'*		C24H25O13	521.1281	521.1289	-1.64	5.39	284
**naringenin 4' methyl ether (isosakuranetin)**	**2**	(+TaOMT8)	C16H15O5	287.0914	287.0914	-0.11	8.98	288
isosakuranetin (glycoside)	2'		C22H25O10	449.1456	449.1442	-3.66	6.45	284
isosakuranetin (malonyl-glycoside)	2'*		C25H27O13	535.1446	535.1436	-1.99	7.06	284
**naringenin 4',7-dimethyl ether**	**3**	(+TaOMT8+TaOMT3)	C17H17O5	301.1070	301.1062	-2.72	11.24	288
**2',5-dihydroxy-4',7-dimethoxyflavanone (artocarpanone A)**	**4**	(+TaOMT8+TaOMT3+TaCYP71C164)	C17H17O6	317.1019	317.1010	-3.10	9.81	288
Artocarpanone A (glycoside)	4'		C23H27O11	479.1547	479.1537	-2.24	7.46	288
Artocarpanone A (diglycoside)	4"		C29H37O16	641.2076	641.2058	-2.81	6.42	288
Artocarpanone A (malonyl-glycoside)	4'*		C26H29O14	565.1551	565.1539	-2.32	8.04	288
**2′,5-dihydroxy-4′,7-dimethoxyisoflavone (2'-*O*-demethyl-triticein)**	**5**	(+TaOMT8+TaOMT3+TaCYP71C164+TaCYP71F53)	C17H15O6	315.0863	315.0855	-2.55	9.63	260
2'-demethyl-triticein (glycoside)	5'		C23H25O11	477.1391	477.1380	-2.45	7.05	260
2'-demethyl-triticein (diglycoside)	5"		C29H35O16	639.1919	639.1906	-2.21	6.11	260
2'-demethyl-triticein (malonyl-glycoside)	5'*		C26H27O14	563.1395	563.1386	-1.64	7.68	260
**5-hydroxy-2′,4′,7-trimethoxyisoflavone (triticein)**	**6**	(+TaOMT8+TaOMT3+TaCYP71C164+TaCYP71F53+TaOMT6)	C18H17O6	329.1019	329.1012	-2.33	11.08	260
triticein (glycoside)	6'		C24H27O11	491.1547	491.1537	-2.30	6.83	260
triticein (diglycoside)	6"		C30H37O16	653.2076	653.2062	-2.10	5.91	260
**naringenin trimethyl ether**	**7**	(+TaOMT8+TaOMT3+TaOMT6)	C18H19O5	315.1227	315.1221	-2.00	9.00	284
**3,5-dihydroxy-4',7-dimethoxyflavanone**	**8**	(+TaOMT8+TaOMT3+TaCYP71F53)	C17H17O6	317.1019	317.1012	-2.33	9.06	292
3,5-dihydroxy-4',7-dimethoxyflavanone (glycoside)	8'		C23H27O11	479.1547	479.1535	-2.62	5.92	284
3,5-dihydroxy-4',7-dimethoxyflavanone (diglycoside)	8"		C29H37O16	641.2076	641.2062	-2.24	4.99	284
3,5-dihydroxy-4',7-dimethoxyflavanone (methylated)	8^		C18H19O6	331.1176	331.1164	-3.78	8.05	288
**2',4',5,7-tetramethoxyflavanone**	**9**	(+TaOMT8+TaOMT3+TaCYP71C164+TaOMT6)	C19H21O6	345.1332	345.1324	-2.46	9.34	284

Table S2. Compounds identified in this study. Rt PDA, retention time on photo diode array chromatogram. NMR spectra for compounds (**4**), (**5**), (**6**), (**8**) are provided in Supplementary Data 1.

Full CDS (Gateway) cloning

TaCHS1 F	**GGGGACAAGTTTGTACAAAAAAGCAGGCT**ATGGCCGCCGCTGTTACCGTAGACGAAG
TaCHS1 R	**GGGGACCACTTTGTACAAGAAAGCTGGGT**TCAAGCGGCTGTTGTCCTCACACTG
chi-D1 F	**GGGGACAAGTTTGTACAAAAAAGCAGGCT**ATGGCAGTTTCCGAACTAGAAGTTG
chi-D1 R	**GGGGACCACTTTGTACAAGAAAGCTGGGT**TCATACTGAGACACTGACTGCTTCA
TaOMT8 F	**GGGGACAAGTTTGTACAAAAAAGCAGGCT**ATGGGTTCTATCTCCGACGACGAGGCG
TaOMT8 R	**GGGGACCACTTTGTACAAGAAAGCTGGGT**CTATTTGTTCAACTCAATGACCCATATG
TaOMT3 F	**GGGGACAAGTTTGTACAAAAAAGCAGGCT**ATGGGCTCCACTGCCGTGGAGAAGGTC
TaOMT3 R	**GGGGACCACTTTGTACAAGAAAGCTGGGT**CTATTTGACGAACTCAATGACCCATGC
TaOMT6 F	**GGGGACAAGTTTGTACAAAAAAGCAGGCT**ATGGAAGATCTCGTGAAGAAACC
TaOMT6 R	**GGGGACCACTTTGTACAAGAAAGCTGGGT**TCAAGGGTAGAGCTCAATAACAGATCTAACTC
TaOMT7 F	**GGGGACAAGTTTGTACAAAAAAGCAGGCT**ATGGCGCCCACCCAAGGCAAGCAGAGTTCTCAGGA
TaOMT7 R	**GGGGACCACTTTGTACAAGAAAGCTGGGT**TCAAGGGTAGAGCTCAATAACAGATCTAAC
TaCYP71C164 F	**GGGGACAAGTTTGTACAAAAAAGCAGGCT**ATGGAAGATCTCGTGAAGAAACC
TaCYP71C164 R	**GGGGACCACTTTGTACAAGAAAGCTGGGT**CTACATCCAGGATTTTGGAATTAACAATAG
TaCYP71F53 F	**GGGGACAAGTTTGTACAAAAAAGCAGGCT**ATGGAGGGTTGGTTAACCTTATGTTTC
TaCYP71F53 R	**GGGGACCACTTTGTACAAGAAAGCTGGGT**CTATATAGTGGAGCGTACATATGGAATAGC
AtMYB12 F	**GGGGACAAGTTTGTACAAAAAAGCAGGCT**ATGGGAAGAGCGCCATGTTGCGAG
AtMYB12 R	**GGGGACCACTTTGTACAAGAAAGCTGGGT**TCATGACAGAAGCCAAGCGACCAAAGC

***T. aestivum* qRT-PCR**

TaCHS1 F	ACTGTTCCAACTTGTCTCGG
TaCHS1 R	CACCTTCCACCTCCACAAG
chi-D1 F	GGTGTGAACGAACCGGGG
chi-D1 R	GAAATGGAGGGAACACGACG
TaOMT8 F	TCAACTTCGACCTCCCTCAC
TaOMT8 R	TGCAATGCTCATCGTTCCAG
TaOMT3 F	AGTACAACAGCACAGACCCA
TaOMT3 R	GTCGAAGCCCCTGTAGAACT
TaOMT6 F	AGATGCTGGTGGAAAGGTGA
TaOMT6 R	CCCCTCAGCACCCATGATAA
TaCYP71C164 F	GGGTTCAACGTGGAGGAGTA
TaCYP71C164 R	CCTTGCTCACATGATCGTCG
TaCYP71F53 F	CCAATGACATCGCCTTCTCG
TaCYP71F53 R	GCTCCATGATGCAGACCTTG
TUBB F	CAAGGAGGTGGACGAGCAGATG
TUBB R	GACTTGACGTTGTTGGGGATCCA

KASP genotyping

Cad0227 (5D:522761619 C/T)	
FAM	**GAAGGTGACCAAGTTCATGCT**AGCTGCGGTCATGGAGGGTTGg
HEX/VIC:	**GAAGGTCGGAGTCAACGGATT**AGCTGCGGTCATGGAGGGTTGa
common:	TTGCTCTTGCCACCGGAAAA
Cad1793 (5D:522759509 G/A)	
FAM:	**GAAGGTGACCAAGTTCATGCT**GATCGCTATTGCCAATGATAGCTCg
HEX/VIC:	**GAAGGTCGGAGTCAACGGATT**GATCGCTATTGCCAATGATAGCTCa
common:	GGTTCGAGAGGTACTGGGTCAT
Cad1682 (5D:524249622 C/T)	
FAM:	**GAAGGTGACCAAGTTCATGCT**TGGCCTTCGACGGAAGCTg
HEX/VIC:	**GAAGGTCGGAGTCAACGGATT**TGGCCTTCGACGGAAGCTa
common:	AATGACCCTCAAGAACGCCATC
Cad1684 (5D:524260382 G/A)	
FAM:	**GAAGGTGACCAAGTTCATGCT**GTCGGTAAATGTTTTAAAGg
HEX/VIC:	**GAAGGTCGGAGTCAACGGATT**GTCGGTAAATGTTTTAAAGa
common:	GTAATCAAACGCTGTCATTC

Table S3. Oligos used in this study.

REFERENCES

1 Ma, S. *et al.* WheatOmics: A platform combining multiple omics data to accelerate functional genomics studies in wheat. *Mol Plant* **14**, 1965-1968 (2021). https://doi.org/10.1016/j.molp.2021.10.006

2 Palma-Guerrero, J. *et al.* Comparative transcriptomic analyses of Zymoseptoria tritici strains show complex lifestyle transitions and intraspecific variability in transcription profiles. *Mol Plant Pathol* **17**, 845-859 (2016). https://doi.org/10.1111/mpp.12333

3 Zhang, H. *et al.* Large-scale transcriptome comparison reveals distinct gene activations in wheat responding to stripe rust and powdery mildew. *BMC Genomics* **15**, 898 (2014). https://doi.org/10.1186/1471-2164-15-898

4 Hofstad, A. N. *et al.* Examining the Transcriptional Response in Wheat Fhb1 Near-Isogenic Lines to Fusarium graminearum Infection and Deoxynivalenol Treatment. *Plant Genome* **9** (2016). https://doi.org/10.3835/plantgenome2015.05.0032

5 Ramirez-Gonzalez, R. H. *et al.* The transcriptional landscape of polyploid wheat. *Science* **361**, 662-+ (2018). https://doi.org/10.1126/science.aar6089

6 Garcia-Seco, D. *et al.* Transcriptome and proteome analysis reveal new insight into proximal and distal responses of wheat to foliar infection by Xanthomonas translucens. *Sci Rep* **7**, 10157 (2017). https://doi.org/10.1038/s41598-017-10568-8

7 Winter, D. *et al.* An "Electronic Fluorescent Pictograph" browser for exploring and analyzing large-scale biological data sets. *PLoS One* **2**, e718 (2007). https://doi.org/10.1371/journal.pone.0000718

8 Polturak, G. *et al.* Pathogen-induced biosynthetic pathways encode defense-related molecules in bread wheat. *Proc Natl Acad Sci U S A* **119**, e2123299119 (2022). https://doi.org/10.1073/pnas.2123299119

9 Kumar, S., Stecher, G. & Tamura, K. MEGA7: Molecular Evolutionary Genetics Analysis Version 7.0 for Bigger Datasets. *Molecular Biology and Evolution* **33**, 1870-1874 (2016). https://doi.org/10.1093/molbev/msw054

10 Arora, S. *et al.* A wheat kinase and immune receptor form host-specificity barriers against the blast fungus. *Nat Plants* **9**, 385-392 (2023). https://doi.org/10.1038/s41477-023-01357-5

11 Katoh, K., Misawa, K., Kuma, K. & Miyata, T. MAFFT: a novel method for rapid multiple sequence alignment based on fast Fourier transform. *Nucleic Acids Res* **30**, 3059-3066 (2002). https://doi.org/10.1093/nar/gkf436

12 Price, M. N., Dehal, P. S. & Arkin, A. P. FastTree 2--approximately maximum-likelihood trees for large alignments. *PLoS One* **5**, e9490 (2010). https://doi.org/10.1371/journal.pone.0009490

13 Stamatakis, A. RAxML version 8: a tool for phylogenetic analysis and post-analysis of large phylogenies. *Bioinformatics* **30**, 1312-1313 (2014). https://doi.org/10.1093/bioinformatics/btu033
