# Supplementary material for: Discovery of isoflavone phytoalexins in wheat reveals an alternative route to isoflavonoid biosynthesis
Source: Nat Commun. 2023 Nov 1;14:6977. doi: 10.1038/s41467-023-42464-3 (PMC10620232; doi:10.1038/s41467-023-42464-3)
Supplement: Supplementary file 3 — Description of Additional Supplementary Files [file 41467_2023_42464_MOESM3_ESM.pdf]

Description of Additional Supplementary Files

Supplementary Data 1. Summary of compound purification procedures and NMR analyses. Compound purification procedures and detailed NMR analyses are given for triticein, 3,5-dihydroxy-4',7-dimethoxyflavanone ,2'-*O*-demethyl triticein, and artocarpanone A.

Supplementary Data 2. Summary of variants in wheat TILLING lines used in this study. All variants are shown for wheat 'Cadenza' TILLING lines Cad1682, Cad1684, Cad1793, Cad0227, together with a sheet including only high impact variants of the four lines, and a sheet presenting only high impact variants found in relevant genes (cytochrome P450s and *O*-methyltransferases). SIFT score predicts whether an amino acid substitution is likely to affect protein function. Substitutions with a score < 0.05 are defined as deleterious.

Supplementary Data 3. Summary of BGC4(5D) Blastn analysis in wheat relatives. Blastn results used for construction of Figure 5b are shown separately for each species analyzed, including *T. aestivum* (subgenomes A/B), *T. turgidum ssp. dicoccoides, T. turgidum ssp. durum, A. tauschii, A. bicornis, A. longissima, A. speltoides, A. sharonensis, A. searsii, H. vulgare, B. distachyon*.
