# Supplementary material for: Discovery of isoflavone phytoalexins in wheat reveals an alternative route to isoflavonoid biosynthesis
Source: Nat Commun. 2023 Nov 1;14:6977. doi: 10.1038/s41467-023-42464-3 (PMC10620232; doi:10.1038/s41467-023-42464-3)
Supplement: Supplementary file 4 — Supplementary Data 1 [file 41467_2023_42464_MOESM4_ESM.pdf]

Discovery of Isoflavone Phytoalexins in Wheat Reveals an Alternative Route to Isoflavonoid Biosynthesis

Supplementary Data 1

Table of Contents

General procedures for compound purification and NMR analyses 3

Purification of 5-hydroxy-2',4',7-trimethoxyisoflavone (triticein) 3

Purification of 3,5-dihydroxy-4',7-dimethoxyflavanone 3

Purification of 2',5-dihydroxy-4',7-dimethoxyisoflavone (2'-*O*-demethyl triticein) 4

Purification of 2',5-dihydroxy-4',7-dimethoxyflavanone (Artocarpanone A) 4

5-hydroxy-2',4',7-trimethoxyisoflavone (triticein) 5

Table 1: ^{1}H, ^{13}C NMR spectroscopic data reported for 5-hydroxy-2',4',7-trimethoxyisoflavone 5

Figure 1: Key ^{1}H-^{1}H COSY (**bold blue**), ^{1}H-^{13}C HMBC (H→C, red arrows) and ^{1}H-^{1}H ROESY (H↔H, green) for 5-hydroxy-2',4',7-trimethoxyisoflavone 6

Figure 2: ^{1}H-NMR spectrum of 5-hydroxy-2',4',7-trimethoxyisoflavone in DMSO-d_6, 600 MHz 7

Figure 3: ^{1}H-^{1}H COSY spectrum of 5-hydroxy-2',4',7-trimethoxyisoflavone in DMSO-d_6, 600 MHz 7

Figure 4: ^{1}H-^{13}C HSQC spectrum of 5-hydroxy-2',4',7-trimethoxyisoflavone in DMSO-d_6, 600 and 150 MHz 8

Figure 5: ^{1}H-^{13}C HMBC spectrum of 5-hydroxy-2',4',7-trimethoxyisoflavone in DMSO-d_6, 600 and 150 MHz 8

Figure 6: ^{1}H-^{1}H ROESY spectrum of 5-hydroxy-2',4',7-trimethoxyisoflavone in DMSO-d_6, 600 MHz 9

Figure 7: DEPTQ-135 spectrum of 5-hydroxy-2',4',7-trimethoxyisoflavone in DMSO-d_6, 150 MHz. 9

3,5-dihydroxy-4',7-dimethoxyflavanone 10

Table 2: ^{1}H, ^{13}C NMR spectroscopic data reported for 3,5-dihydroxy-4',7-dimethoxyflavanone 10

in Acetone-d_6, 600 and 150 MHz 10

Figure 8: Key ^{1}H-^{1}H COSY (**bold blue**), ^{1}H-^{13}C HMBC (H→C, red arrows) and ^{1}H-^{1}H ROESY (H↔H, green) of 3,5-dihydroxy-4',7-dimethoxyflavanone 11

Figure 9: ^{1}H-NMR spectrum of 3,5-dihydroxy-4',7-dimethoxyflavanone in Acetone-d_6, 600 MHz .. 12

Figure 10: ^{1}H-^{1}H COSY spectrum of 3,5-dihydroxy-4',7-dimethoxyflavanone in Acetone-d_6, 600 MHz 12

Figure 11: ^{1}H-^{13}C HSQC spectrum of 3,5-dihydroxy-4',7-dimethoxyflavanone in Acetone-d_6, 600 and 150 MHz 13

Figure 12: ^{1}H-^{13}C HMBC spectrum of 3,5-dihydroxy-4',7-dimethoxyflavanone in Acetone-d_6, 600 and 150 MHz 13

Figure 13: ^{1}H-^{1}H ROESY spectrum of 3,5-dihydroxy-4',7-dimethoxyflavanone in Acetone-d_6, 600 MHz 14

Figure 14: ^{13}C-NMR spectrum of 3,5-dihydroxy-4',7-dimethoxyflavanone in Acetone-d_6, 150 MHz14

2',5-dihydroxy-4',7-dimethoxyisoflavone (2'-*O*-demethyl-triticein) 15

Table 3: ^{1}H, ^{13}C NMR spectroscopic data reported for 2',5-dihydroxy-4',7-dimethoxyisoflavone in Acetone-d_6, 600 and 150 MHz. 15

Figure 15: Key ^{1}H-^{1}H COSY (**bold blue**), ^{1}H-^{13}C HMBC (H→C, red arrows) for 2',5-dihydroxy-4',7-dimethoxyisoflavone 16

Figure 16: ^{1}HNMR spectrum of 2',5-dihydroxy-4',7-dimethoxyisoflavone in Acetone-d_6, 600 and 150 MHz 17

Figure 17: ^{1}H-^{1}H COSY spectrum of 2',5-dihydroxy-4',7-dimethoxyisoflavone in Acetone-d_6, 600 and 150 MHz 17

Figure 18: ^{1}H-^{13}C HSQC spectrum of 2',5-dihydroxy-4',7-dimethoxyisoflavone in Acetone-d_6, 600 and 150 MHz 18

Figure 19: ^{1}H-^{13}C HMBC spectrum of 2',5-dihydroxy-4',7-dimethoxyisoflavone in Acetone-d_6, 600 and 150 MHz 18

Figure 20: DEPTQ-135 spectrum of 2',5-dihydroxy-4',7-dimethoxyisoflavone in Acetone-d_6, 600 and 150 MHz 19

2',5-dihydroxy-4',7-dimethoxyflavanone (artocarpanone A) 20

Table 4: ^{1}H, ^{13}C NMR spectroscopic data reported for 2',5-dihydroxy-4',7-dimethoxyflavanone in Acetone-d_6, 600 and 150 MHz 20

Figure 21: Key ^{1}H-^{1}H COSY (**bold blue**), ^{1}H-^{13}C HMBC (H→C, red arrows) and ^{1}H-^{1}H ROESY (H↔H, green) of 2',5-dihydroxy-4',7-dimethoxyflavanone 21

Figure 22: ^{1}H-NMR of 2',5-dihydroxy-4',7-dimethoxyflavanone in Acetone-d_6, 600 MHz 22

Figure 23: ^{1}H-^{1}H COSY of 2',5-dihydroxy-4',7-dimethoxyflavanone in Acetone-d_6, 600 MHz 22

Figure 24: ^{1}H-^{13}C HSQC of 2',5-dihydroxy-4',7-dimethoxyflavanone in Acetone-d_6, 600 and 150 MHz 23

Figure 25: ^{1}H-^{13}C HMBC of 2',5-dihydroxy-4',7-dimethoxyflavanone in Acetone-d_6, 600 and 150 MHz 23

Figure 26: ^{1}H-^{1}H ROESY of 2',5-dihydroxy-4',7-dimethoxyflavanone in Acetone-d_6, 600 MHz 24

Figure 27: DEPTQ-135 of 2',5-dihydroxy-4',7-dimethoxyflavanone in Acetone-d_6, 150 MHz 24

General procedures for compound purification and NMR analyses

Organic solvents used for extraction and flash chromatography were reagent grade and used directly without further distillation. HPLC mobile phases were prepared using HPLC grade solvents. LC-MS spectral data were recorded on a Q-exactive orbitrap mass spectrometer using Kinetex-XB-C_{18} (50 × 10 mm i.d.; 2.6 μm). 1D and 2D NMR spectra were recorded on a Bruker Avance 600 MHz spectrometer equipped with a BBFO Plus Smart probe and a triple resonance TCI cryoprobe, respectively. The chemical shifts are relative to the residual signal solvent (DMSO-*d*$_6$: δ_H 2.50; δ_C 39.51, or Acetone-*d*$_6$: δ_H 2.05; δ_C 29.84 and 206.26). Preparative and Semi-preparative HPLC was performed on an Agilent 1290 Infinity (II) system using Luna C_{18} columns (250 x 21.2 and 250 × 10 mm i.d. respectively; both 5 μm particle size).

Purification of 5-hydroxy-2',4',7-trimethoxyisoflavone (triticein)

pEAQ::*TaCHS1*, *chi-D1*, *TaOMT3*, *TaOMT8*, *CYP71C164*, *CYP71F53*, *TaOMT6* and *AtMYB12* were transiently co-expressed in 100 *N. benthamiana* plants to yield 86 g of dry leaves. Freeze-dried leaf material was extracted using ethyl acetate (1 L X 5) at ambient temperature. The organic extract was collected and the solvent removed under reduced pressure to yield an intense green viscous material. Next, normal phase flash chromatography was performed on an Isolera Prime system with a Sfar silica D 50 gr column (Biotage). A linear gradient of ethyl acetate /hexane [100/0 up to 50/50] over 30 min and total solvent volume of 2400 mL, gave 12 200 mL subfractions, F1-F12. Subfraction F8 that contained the target molecule was further purified using repetitive C_{18} semi-preparative HPLC using a linear gradient of water/acetonitrile (50/50 up to 0/100) acidified with 0.1% FA over 20 min to yield 12.5 mg of 5-hydroxy-2',4',7-trimethoxyisoflavone as a white powder.

Purification of 3,5-dihydroxy-4',7-dimethoxyflavanone

pEAQ::*TaCHS1*, *chi-D1*, *TaOMT3*, *TaOMT8*, *CYP71F53* and *AtMYB12* were transiently co-expressed in 60 *N. benthamiana* plants to yield 103 g of dry leaves. Freeze dried material was extracted using ethyl acetate using a Büchi Speed extractor E916. The organic extract was collected and dried under reduced pressure to yield an intense green viscous material. Next, normal phase flash chromatography was performed on an Isolera Prime system with a Sfar silica D 50 gr column (Biotage). A linear gradient of ethyl acetate /hexane [100/0 up to 50/50] over 30 min and total solvent volume of 2400 mL, gave 12 200 mL subfractions, F1-F12. Subfraction F7 was further purified using repetitive C_{18} semi-preparative HPLC using a linear gradient of water/acetonitrile (50/50 up to 0/100), 4 mL/min and acidified with 0.1% FA over 20 min to finally yield 6.6 mg of 3,5-dihydroxy-4',7-dimethoxyflavanone in the form of pale-yellow needles.

Purification of 2',5-dihydroxy-4',7-dimethoxyisoflavone (2'-*O*-demethyl triticein)

pEAQ::*TaCHS1*, *chi-D1*, *TaOMT3*, *TaOMT8*, *CYP71C164*, *CYP71F53* and *AtMYB12* were transiently co-expressed in 100 *N. benthamiana* plants to yield 50 g of dry leaves. Freeze dried material was defatted by hexane partitioning and extracted with ethyl acetate using a Büchi Speed extractor E916. The organic extract was collected and dried under reduced pressure to yield an intense green viscus material. Next, normal phase flash chromatography was performed on an Isolera Prime system with a Sfar silica D 50 gr column (Biotage). A linear gradient of ethyl acetate /hexane [100/0 up to 0/100] over 30 min and total solvent volume of 2400 mL, gave 12 200 mL subfractions, F1-F12. Subfractions F4 and F5 which contained the target molecule were combined and introduced to repetitive C_{18} preparative HPLC using a linear gradient of water/ACN + 0.1% FA (80/20 up to 0/100) over 22 min. Semi-pure fractions were collected, combined and further purified by semi-preparative HPLC using an isocratic gradient of water/acetonitrile (33/77) acidified with 0.1% FA, 4 mL/min over 10 min to yield 2.65 mg of 2',5-dihydroxy-4',7-dimethoxyisoflavone as a white powder.

Purification of 2',5-dihydroxy-4',7-dimethoxyflavanone (Artocarpanone A)

pEAQ::*TaCHS1*, *chi-D1*, *TaOMT3*, *TaOMT8*, *CYP71C164* and *AtMYB12* were transiently co-expressed in 60 *N. benthamiana* plants to yield 101 g of dry leaves. Freeze dried material was extracted with ethyl acetate using a Büchi Speed extractor E916. The organic extract was collected and dried under reduced pressure to yield an intense green viscus material. Next, normal phase flash chromatography was performed on an Isolera Prime system with a Sfar silica D 50 gr column (Biotage). A linear gradient of ethyl acetate /hexane [100/0 up to 50/50] over 30 min and total solvent volume of 2400 mL, gave 12 200 mL subfractions, F1-F12. Subfraction F6 was further purified using repetitive C_{18} semi-preparative HPLC using a linear gradient of water/acetonitrile [50/50 up 0/100], 4 mL/min and acidified with 0.1% FA over 20 min to finally yield 2.2 mg of 2',5-dihydroxy-4',7-dimethoxyflavanone (Artocarpanone A) as a pale-yellow powder.

5-hydroxy-2’,4’,7-trimethoxyisoflavone (triticein)

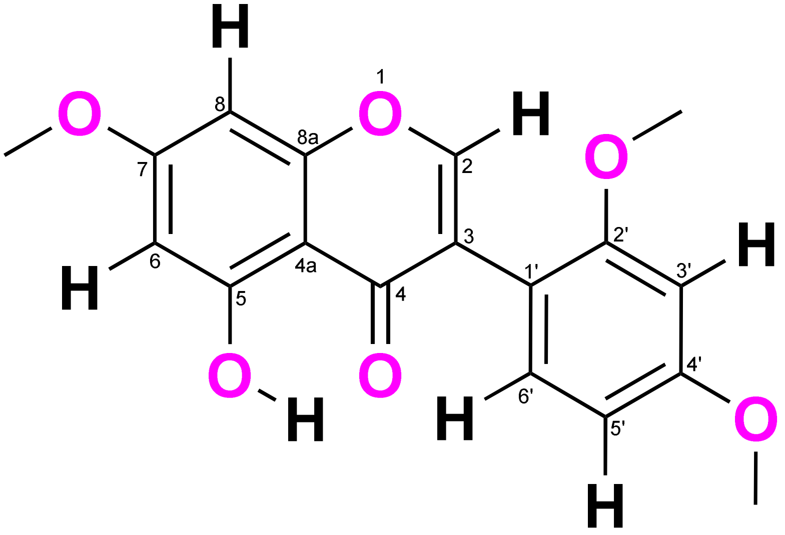

Table 1: ^{1}H, ^{13}C NMR spectroscopic data reported for 5-hydroxy-2’,4’,7-trimethoxyisoflavone (DMSO-d_6) 600 and 150 MHz. Spectra were previously reported[1].

Position	δ_C, Type	δ_H, mult. (J in Hz)
**2**	155.7, CH	8.25, s
**3**	120.6, Cq	-
**4**	180.3, Cq	-
**4a**	105.3, Cq	-
**5**	161.7, Cq	-
**6**	98.1, CH	6.42, *d* (2.3)
**7**	165.3, Cq	-
**8**	92.6, CH	6.66, *d* (2.3)
**8a**	157.6, Cq	-
**1’**	111.9, Cq	-
**2’**	158.5, Cq	-
**3’**	98.7, CH	6.65, *d* (2.4)
**4’**	161.1, Cq	-
**5’**	104.8, CH	6.58, *dd* (8.3, 2.4)
**6’**	132.2, CH	7.17, *d* (8.3)
**7-OMe**	55.4, CH_3	3.87, *s*
**2’-OMe**	56.2, CH_3	3.72, *s*
**4’-OMe**	55.7, CH_3	3.80, *s*
**5-OH**	-	12.89, *s*

Figure 1: Key ¹H-¹H COSY (**bold blue**), ¹H-¹³C HMBC (H→C, red arrows) and ¹H-¹H ROESY (H↔H, green) for 5-hydroxy-2',4',7-trimethoxyisoflavone

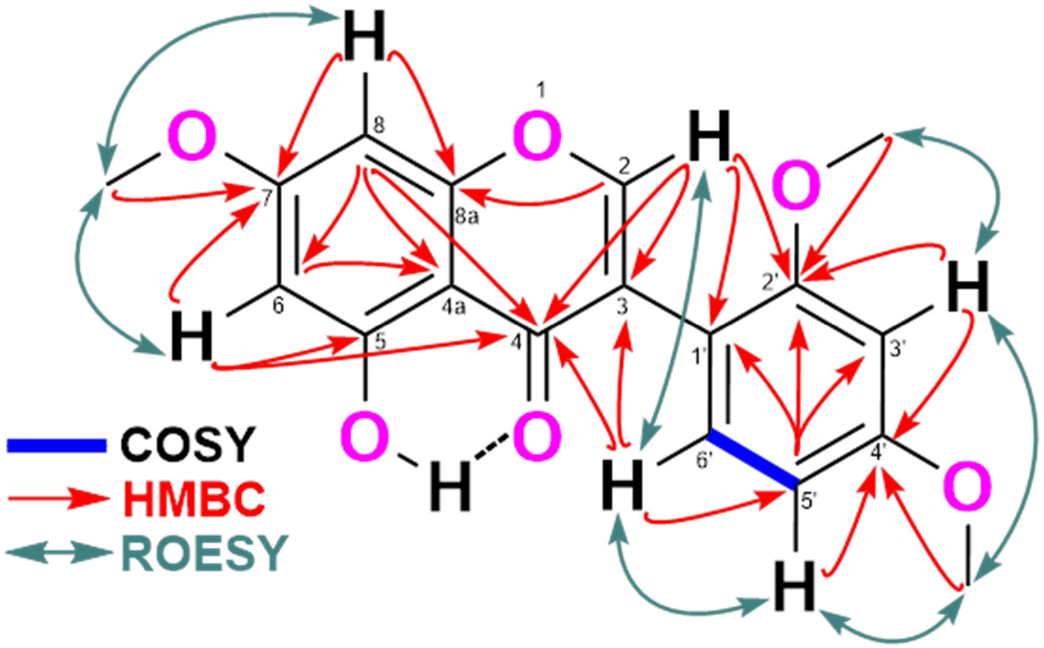

Figure 2: ^{1}H-NMR spectrum of 5-hydroxy-2’,4’,7-trimethoxyisoflavone in DMSO-d_6, 600 MHz

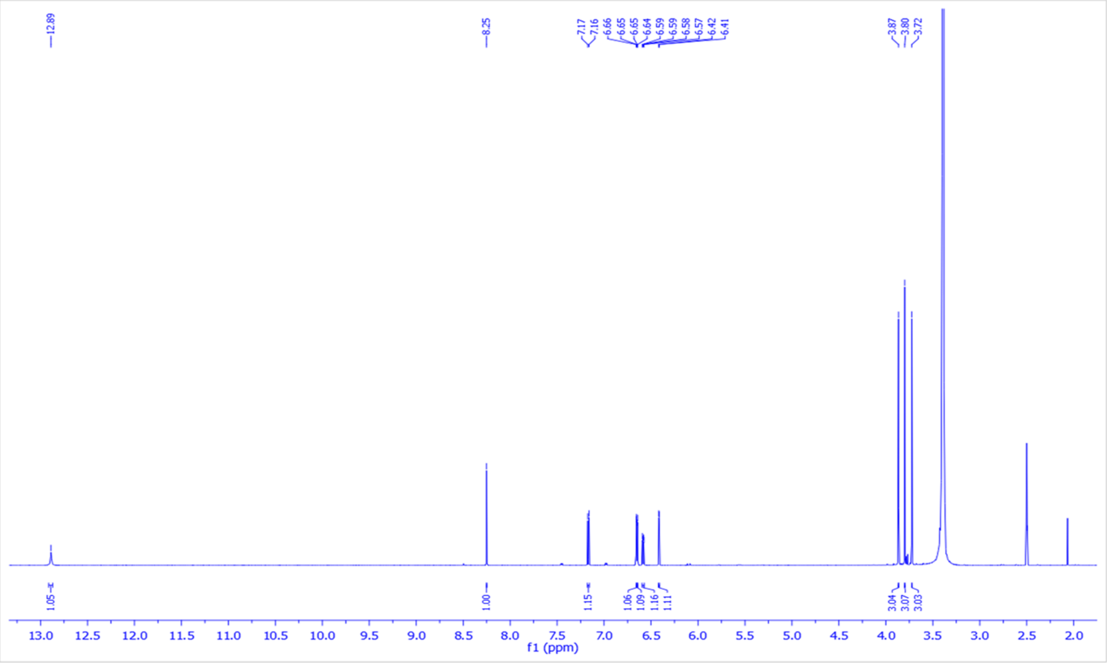

Figure 3: ^{1}H-^{1}H COSY spectrum of 5-hydroxy-2’,4’,7-trimethoxyisoflavone in DMSO-d_6, 600 MHz

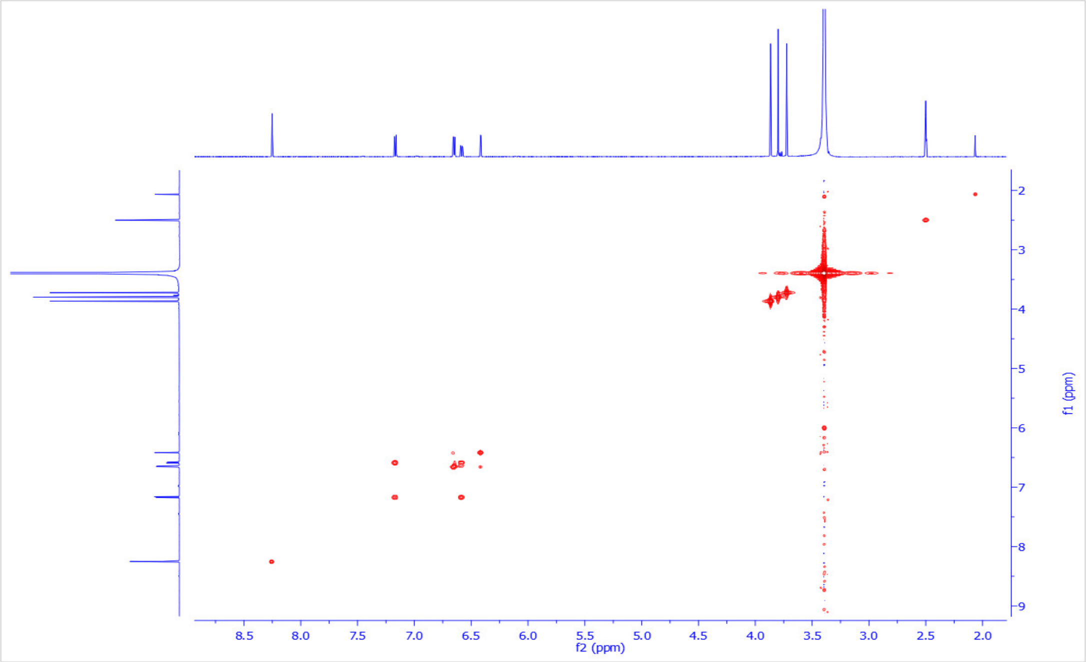

Figure 4: ^{1}H-^{13}C HSQC spectrum of 5-hydroxy-2',4',7-trimethoxyisoflavone in DMSO-d_6, 600 and 150 MHz

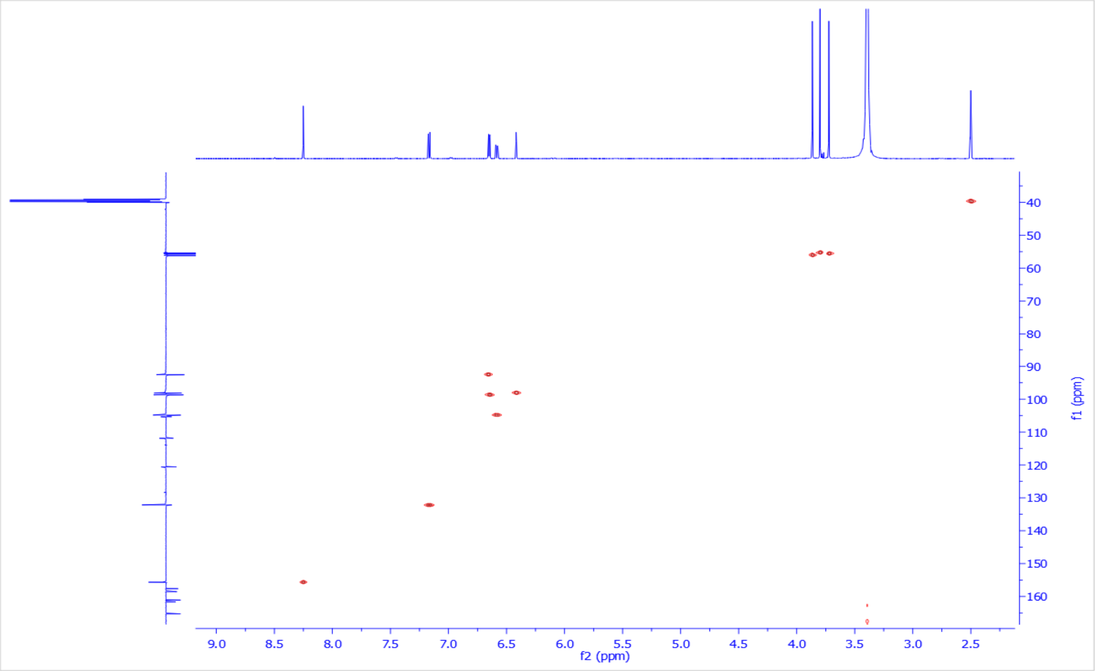

Figure 5: ^{1}H-^{13}C HMBC spectrum of 5-hydroxy-2',4',7-trimethoxyisoflavone in DMSO-d_6, 600 and 150 MHz

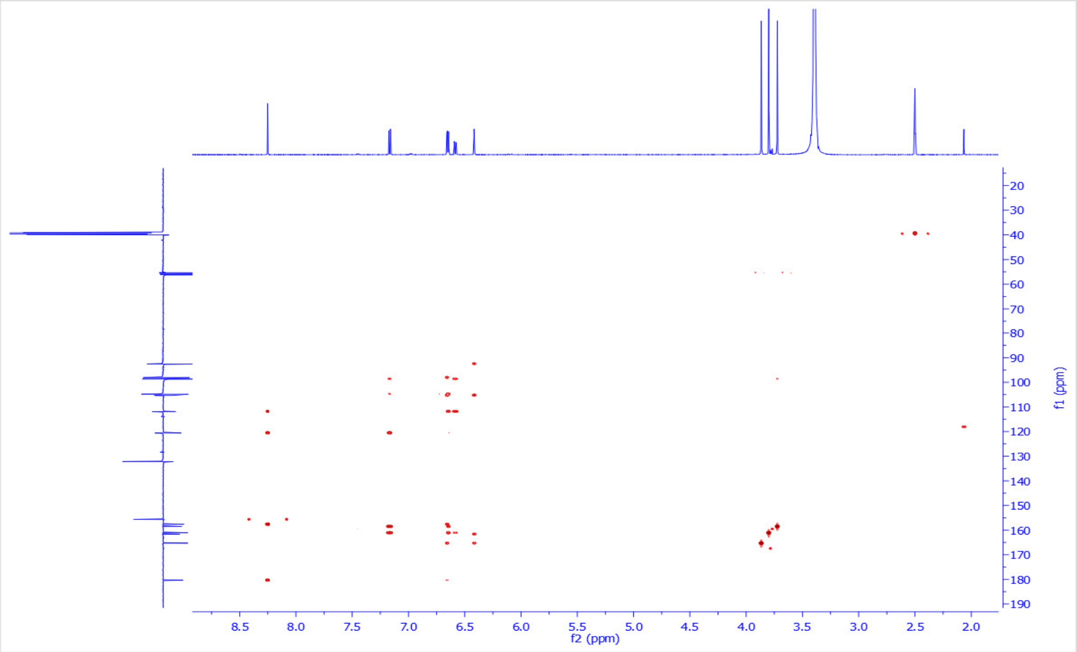

Figure 6: ^{1}H-^{1}H ROESY spectrum of 5-hydroxy-2',4',7-trimethoxyisoflavone in DMSO-d_6, 600 MHz

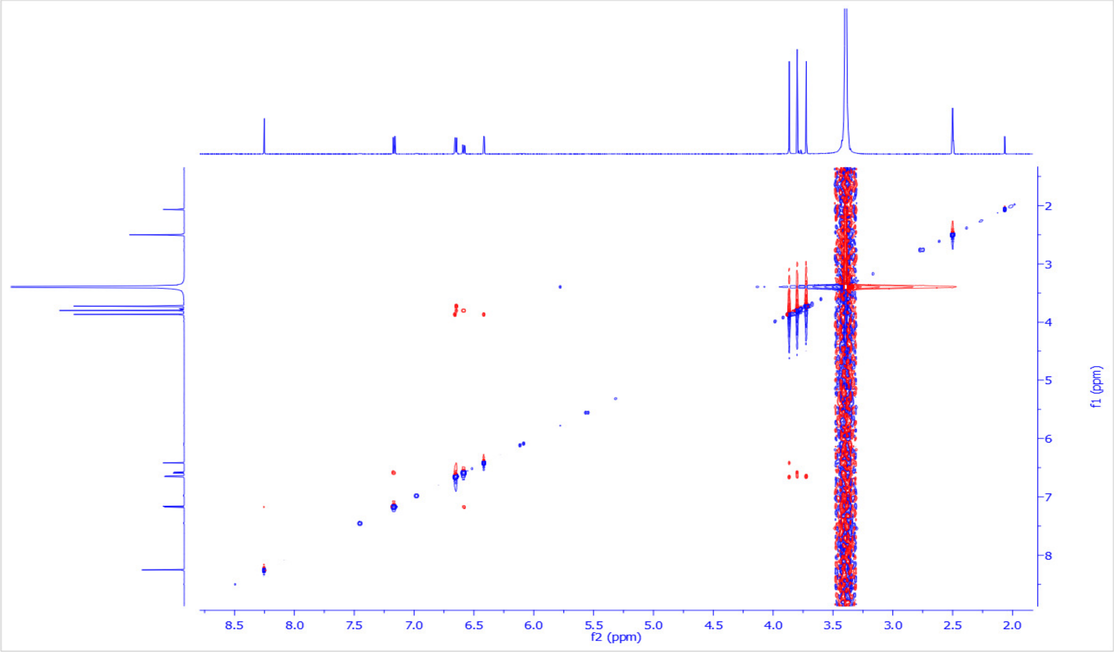

Figure 7: DEPTQ-135 spectrum of 5-hydroxy-2',4',7-trimethoxyisoflavone in DMSO-d_6, 150 MHz

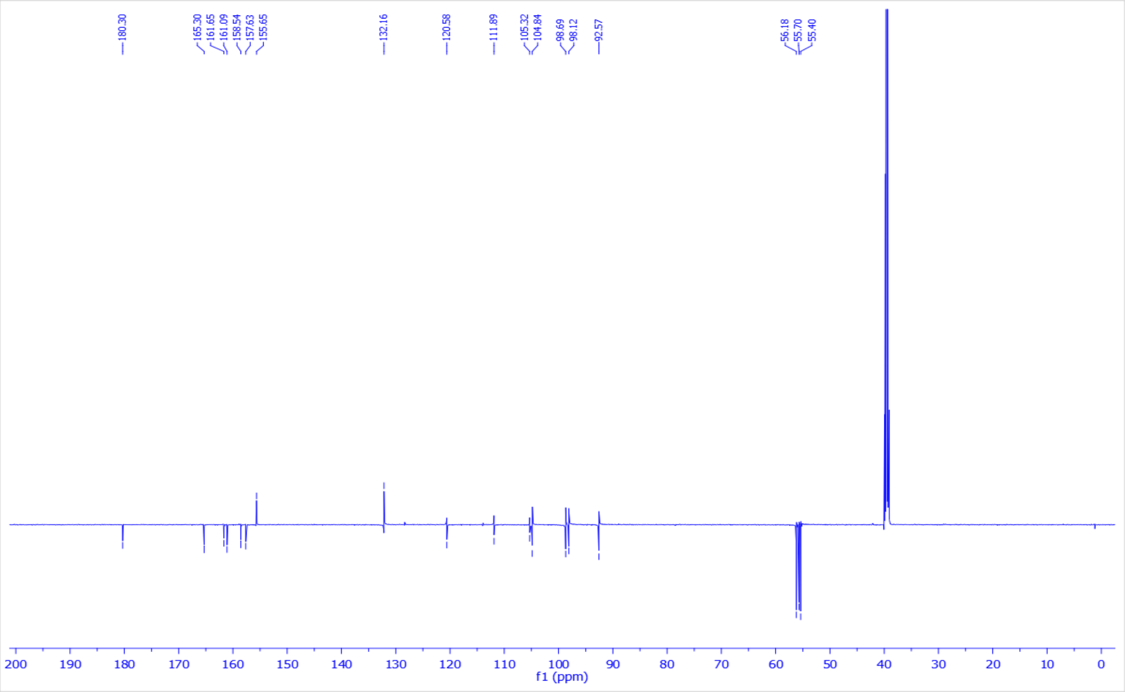

3,5-dihydroxy-4’,7-dimethoxyflavanone

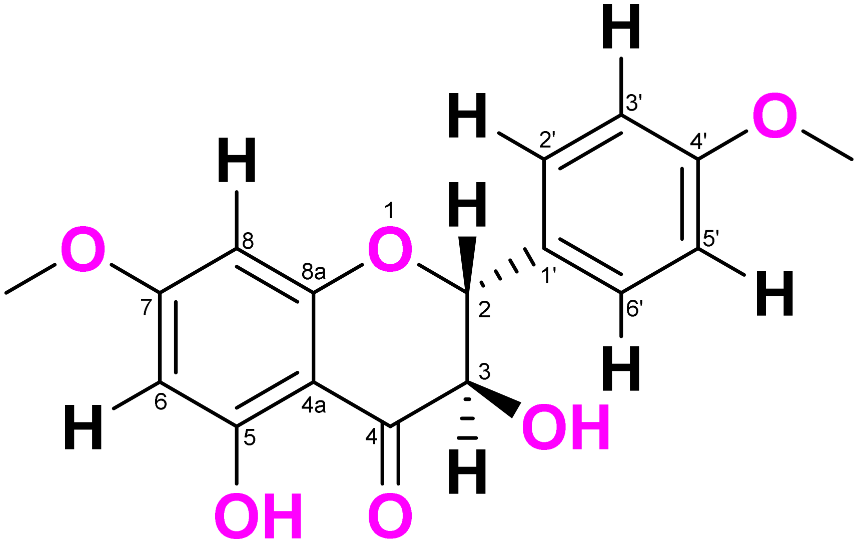

Table 2: ^{1}H, ^{13}C NMR spectroscopic data reported for 3,5-dihydroxy-4’,7-dimethoxyflavanone in Acetone-d_6, 600 and 150 MHz. Spectra were previously reported[2].

Position	δ_C, Type	δ_H, mult. (J in Hz)
**2**	84.3, CH	5.16, *d* (11.6)
**3**	73.2, CH	4.70, *dd* (11.6, 5.8)
**4**	198.6, Cq	-
**4a**	102.1, Cq	-
**5**	164.7, Cq	-
**6**	94.8, CH	6.06, *d* (2.3)
**7**	169.3, Cq	-
**8**	95.8, CH	6.09, *d* (2.3)
**8a**	164.0, Cq	-
**1’**	129.8, Cq	-
**2’**	130.2, CH	7.52, *d* (8.6)
**3’**	114.5, CH	7.00, *d* (8.8)
**4’**	161.2, Cq	-
**5’**	114.5, CH	7.00, *d* (8.8)
**6’**	130.2, CH	7.52, *d* (8.6)
**7-OMe**	56.4, CH_3	3.86, s
**4’-OMe**	55.6, CH_3	3.85, s
**5-OH**	-	11.86, s
**3-OH**	-	4.78, *d* (3.9)

Figure 8: Key ^1H-^1H COSY (**bold blue**), ^1H-^{13}C HMBC (H→C, red arrows) and ^1H-^1H ROESY (H↔H, green) of 3,5-dihydroxy-4',7-dimethoxyflavanone

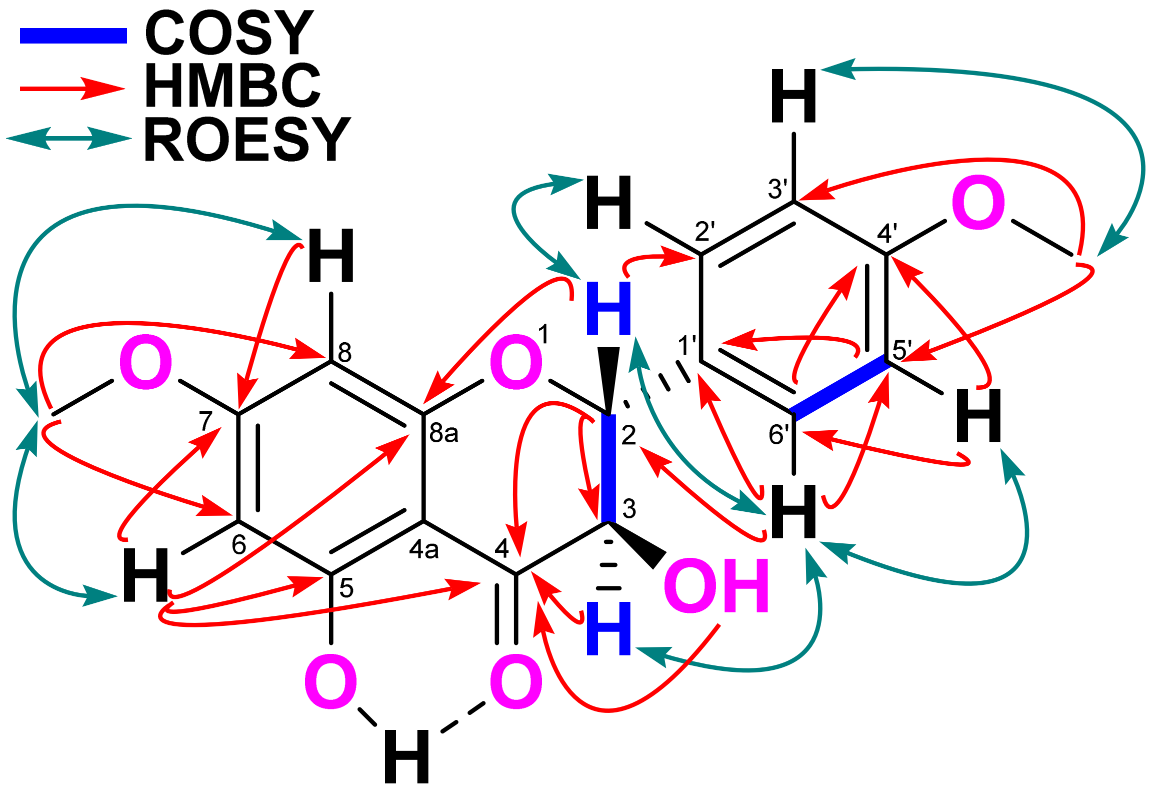

Figure 9: ^{1}H-NMR spectrum of 3,5-dihydroxy-4’,7-dimethoxyflavanone in Acetone-d_6, 600 MHz

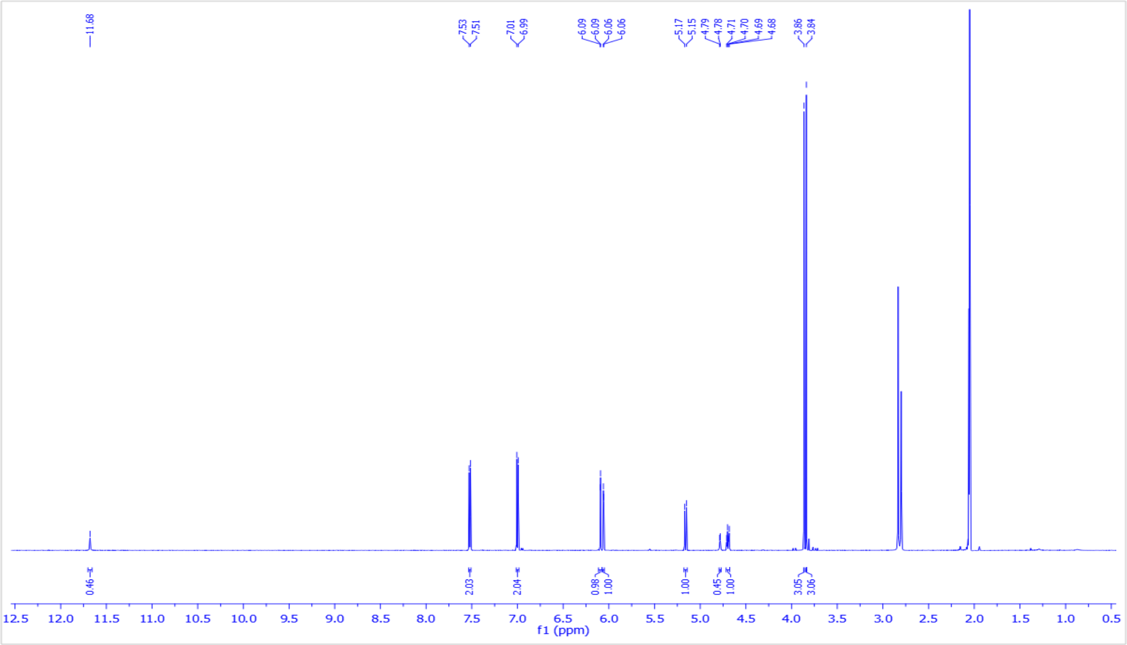

Figure 10: ^{1}H-^{1}H COSY spectrum of 3,5-dihydroxy-4’,7-dimethoxyflavanone in Acetone-d_6, 600 MHz

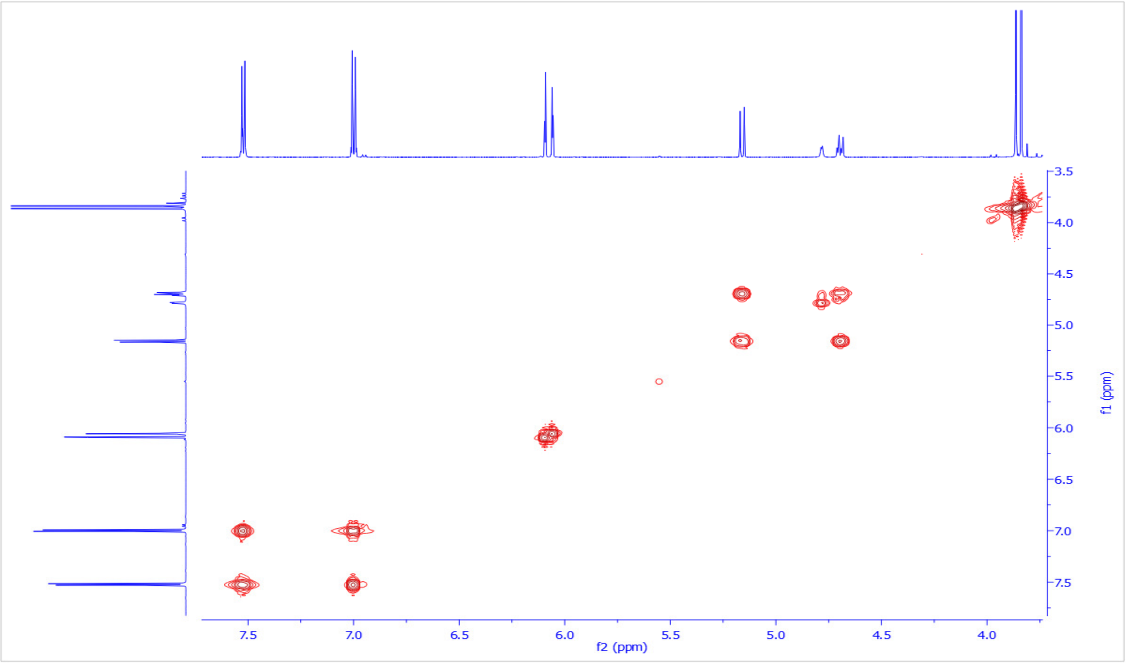

Figure 11: ^{1}H-^{13}C HSQC spectrum of 3,5-dihydroxy-4’,7-dimethoxyflavanone in Acetone-d_6, 600 and 150 MHz

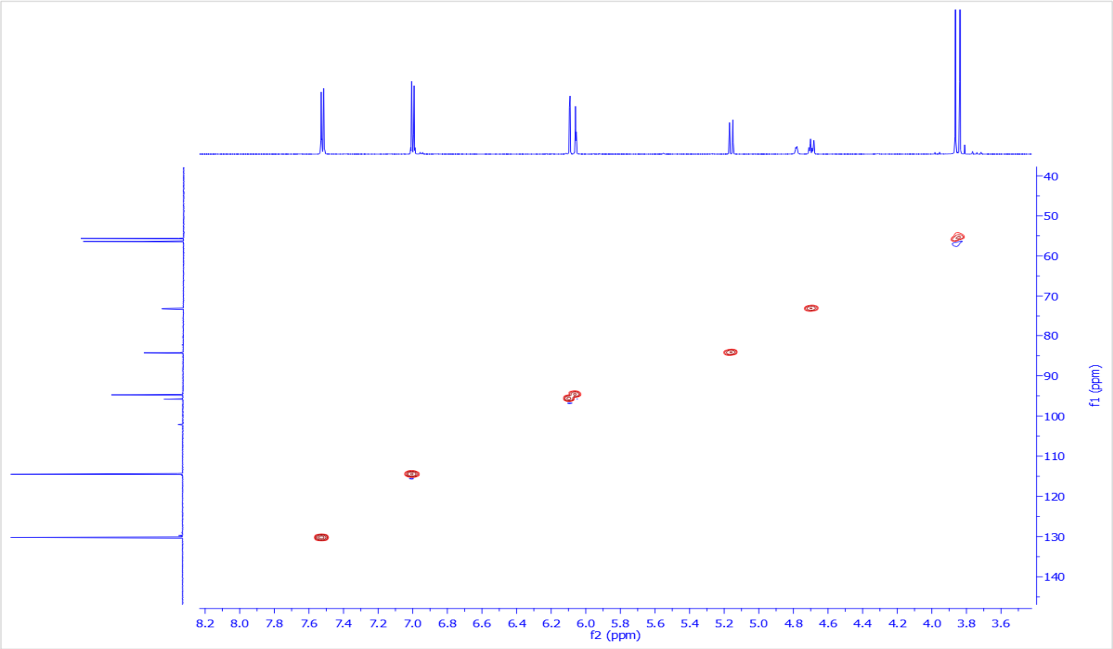

Figure 12: ^{1}H-^{13}C HMBC spectrum of 3,5-dihydroxy-4’,7-dimethoxyflavanone in Acetone-d_6, 600 and 150 MHz

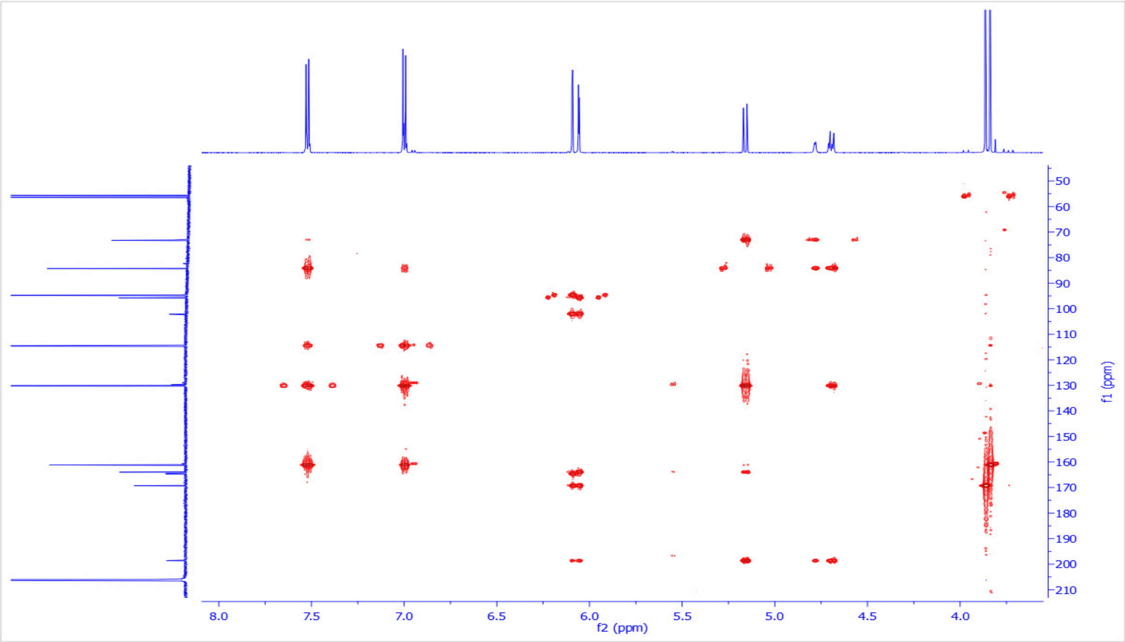

Figure 13: ^{1}H-^{1}H ROESY spectrum of 3,5-dihydroxy-4',7-dimethoxyflavanone in Acetone-d_6, 600 MHz

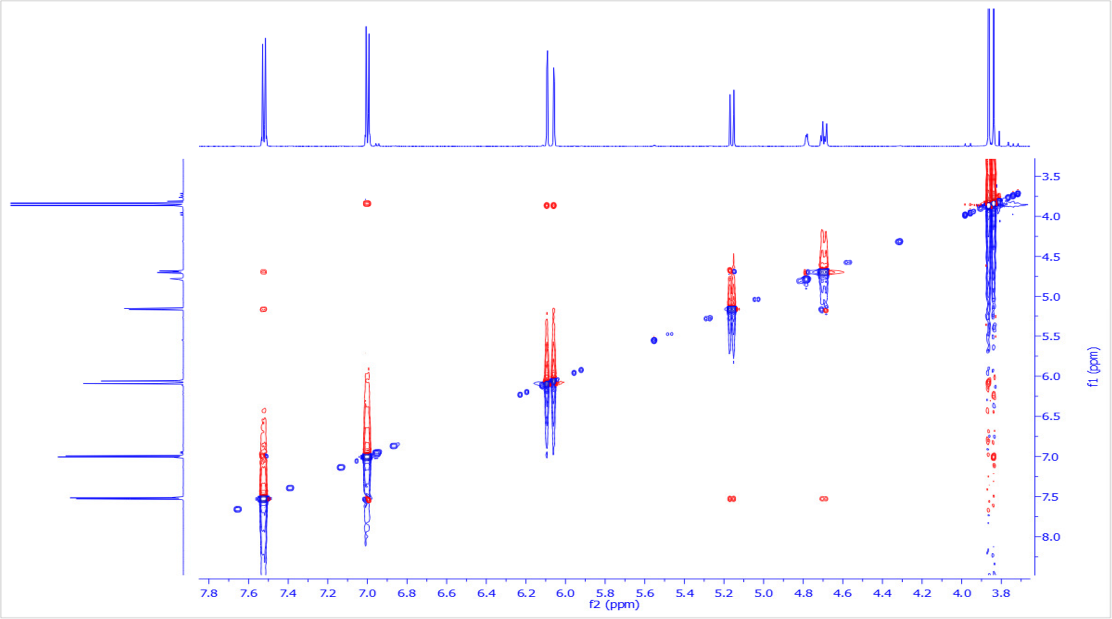

Figure 14: ^{13}C-NMR spectrum of 3,5-dihydroxy-4',7-dimethoxyflavanone in Acetone-d_6, 150 MHz

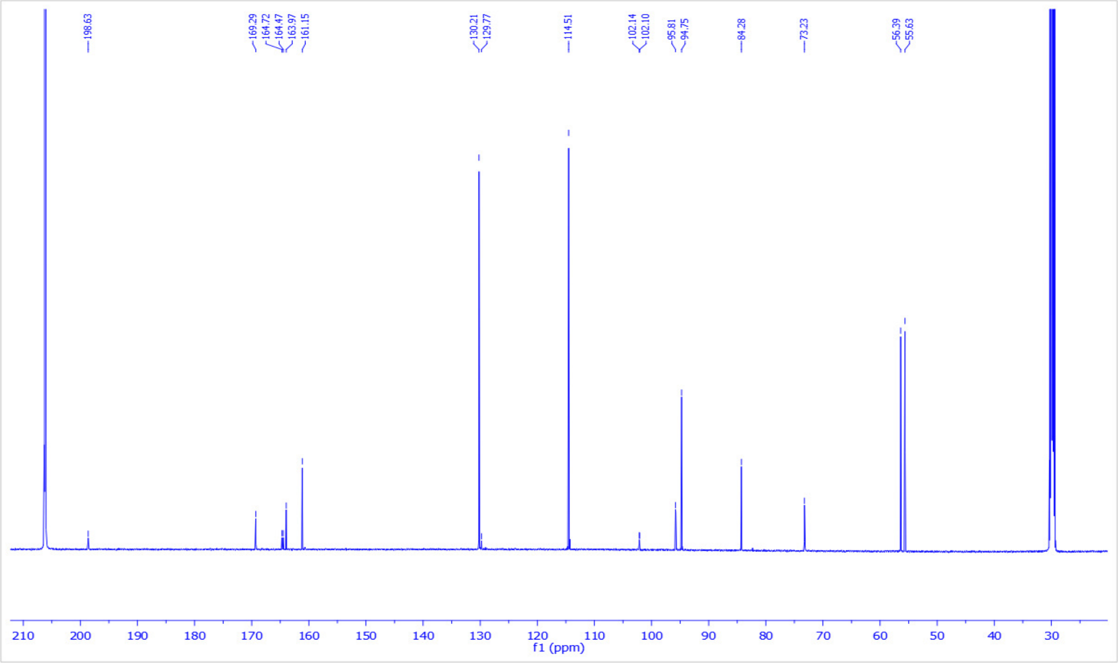

2',5-dihydroxy-4',7-dimethoxyisoflavone (2'-*O*-demethyl-triticein)

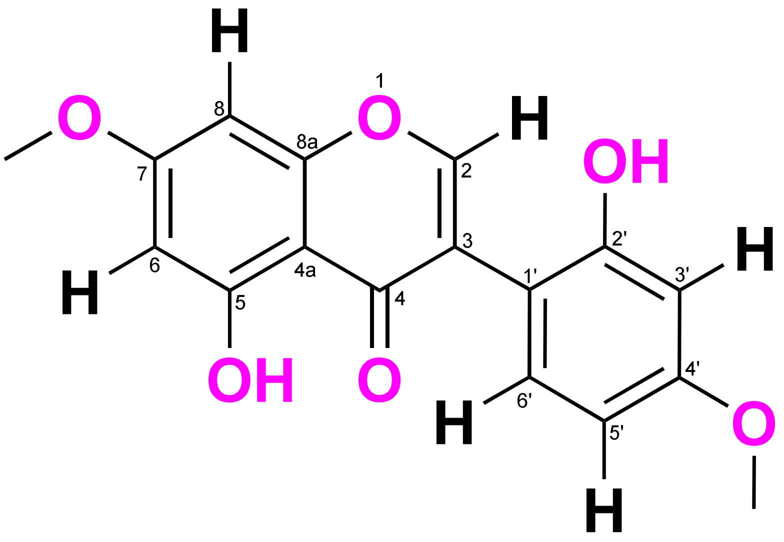

Table 3: ^{1}H, ^{13}C NMR spectroscopic data reported for 2',5-dihydroxy-4',7-dimethoxyisoflavone in Acetone-d_6, 600 and 150 MHz.

Position	δ_C, Type	δ_H, mult. (J in Hz)
**2**	156.9, CH	8.23, s
**3**	122.2, Cq	-
**4**	182.3, Cq	-
**4a**	106.7, Cq	-
**5**	163.4, Cq	-
**6**	99.0, CH	6.39, *d* (2.3)
**7**	166.9, Cq	-
**8**	93.0, CH	6.60, *d* (2.3)
**8a**	159.0, Cq	-
**1'**	111.9, Cq	-
**2'**	157.7, Cq	-
**3'**	103.2, CH	6.54, *d* (2.4)
**4'**	162.4, Cq	-
**5'**	106.6, CH	6.53, *m*
**6'**	133.0, CH	7.22, *d* (8.8)
**7-OMe**	56.5, CH_3	3.94, *s*
**4'-OMe**	55.6, CH_3	3.80, *s*
**2'-OH**	-	8.39, *s*
**5-OH**	-	12.76, *s*

Figure 15: Key ^{1}H-^{1}H COSY (**bold blue**), ^{1}H-^{13}C HMBC (H→C, red arrows) for 2',5-dihydroxy-4',7-dimethoxyisoflavone

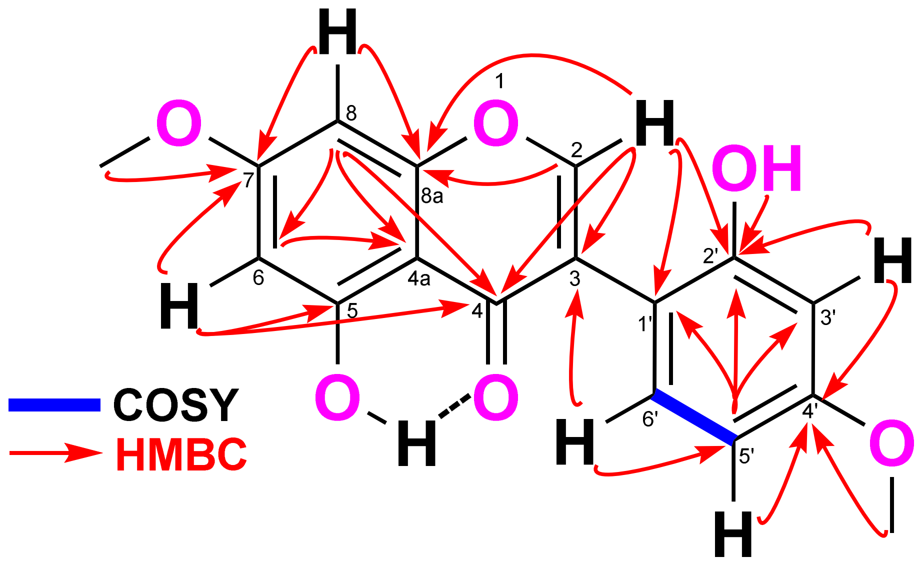

Figure 16: ^{1}HNMR spectrum of 2',5-dihydroxy-4',7-dimethoxyisoflavone in Acetone-d_6, 600 and 150 MHz

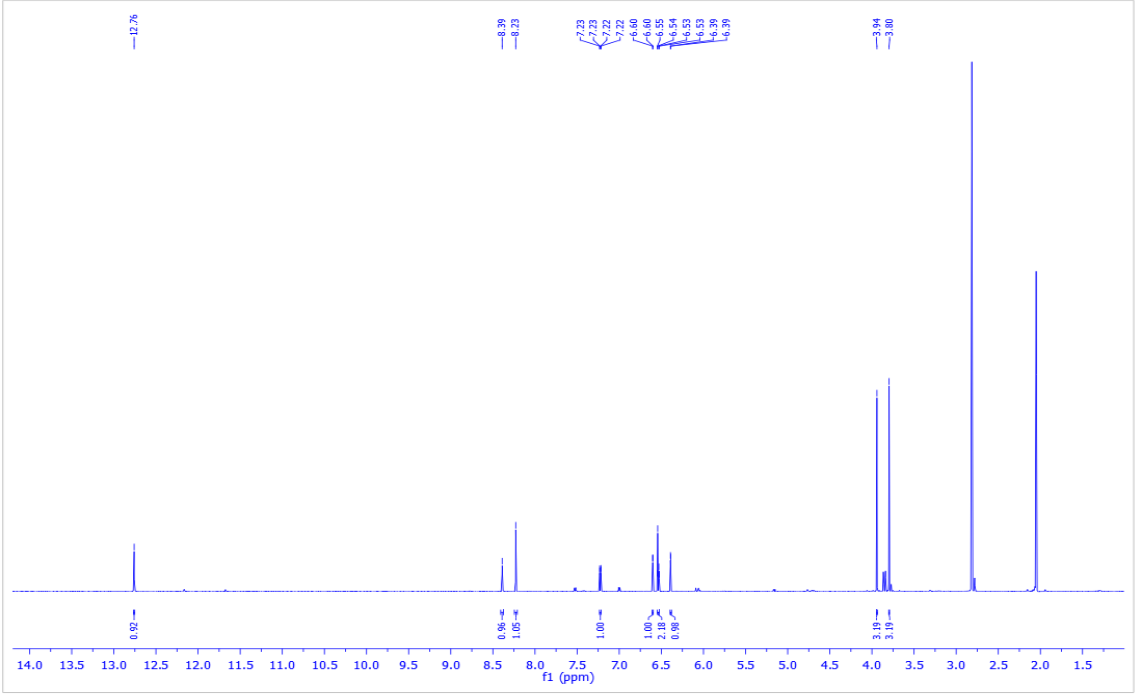

Figure 17: ^{1}H-^{1}H COSY spectrum of 2',5-dihydroxy-4',7-dimethoxyisoflavone in Acetone-d_6, 600 and 150 MHz

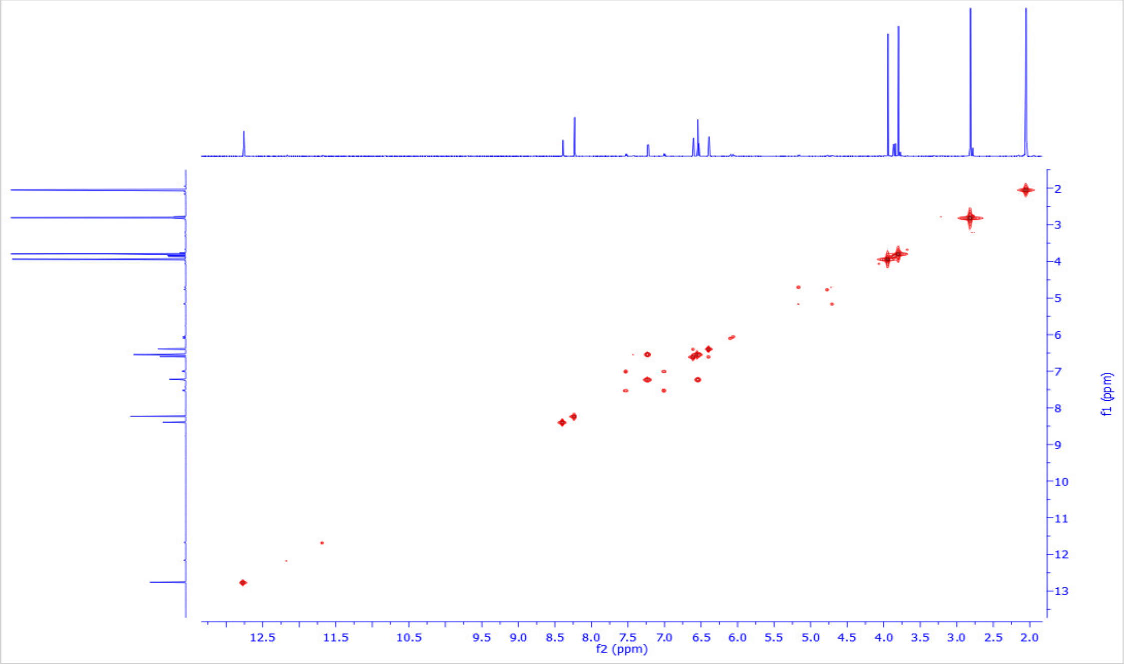

Figure 18: ^{1}H-^{13}C HSQC spectrum of 2',5-dihydroxy-4',7-dimethoxyisoflavone in Acetone-d_6, 600 and 150 MHz

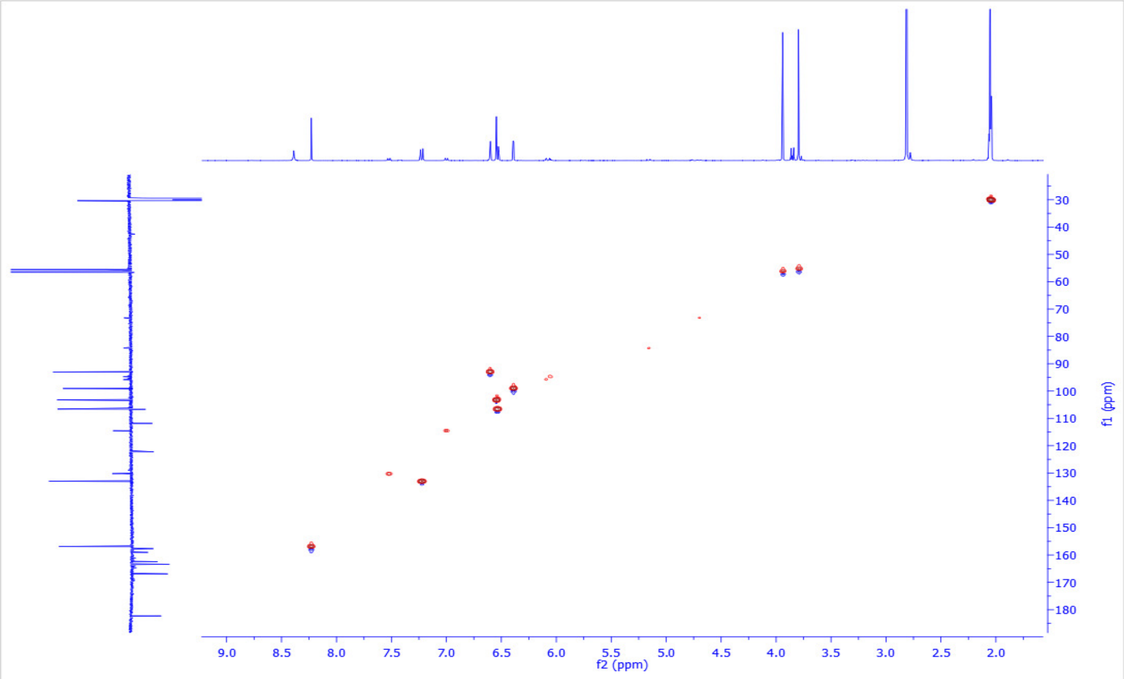

Figure 19: ^{1}H-^{13}C HMBC spectrum of 2',5-dihydroxy-4',7-dimethoxyisoflavone in Acetone-d_6, 600 and 150 MHz

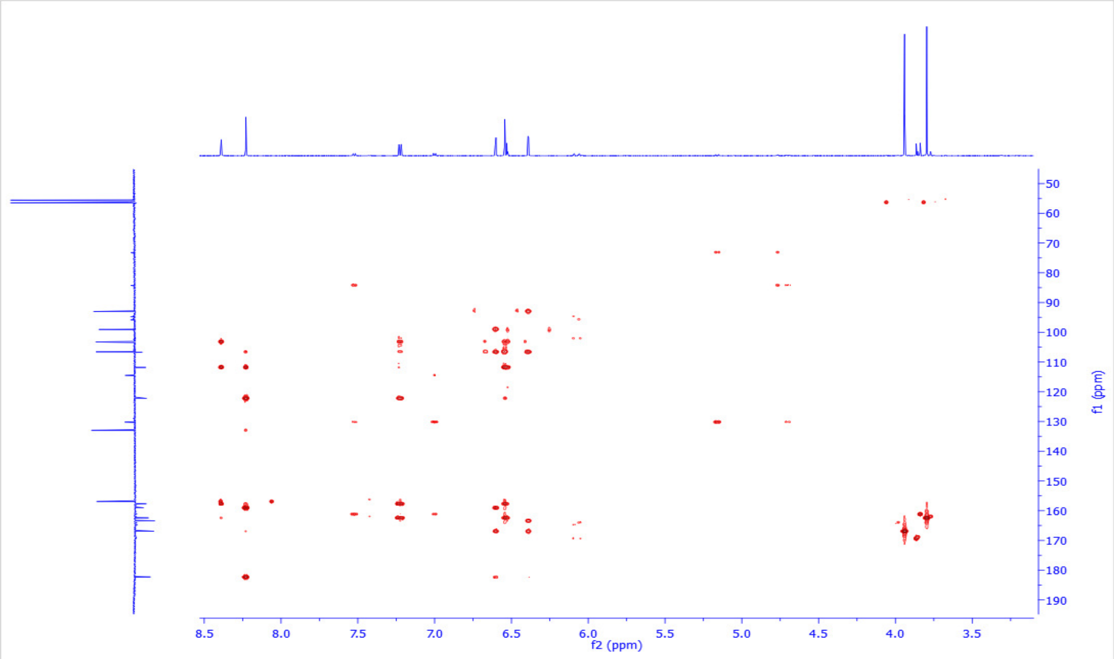

Figure 20: DEPTQ-135 spectrum of 2',5-dihydroxy-4',7-dimethoxyisoflavone in Acetone-d_6, 600 and 150 MHz

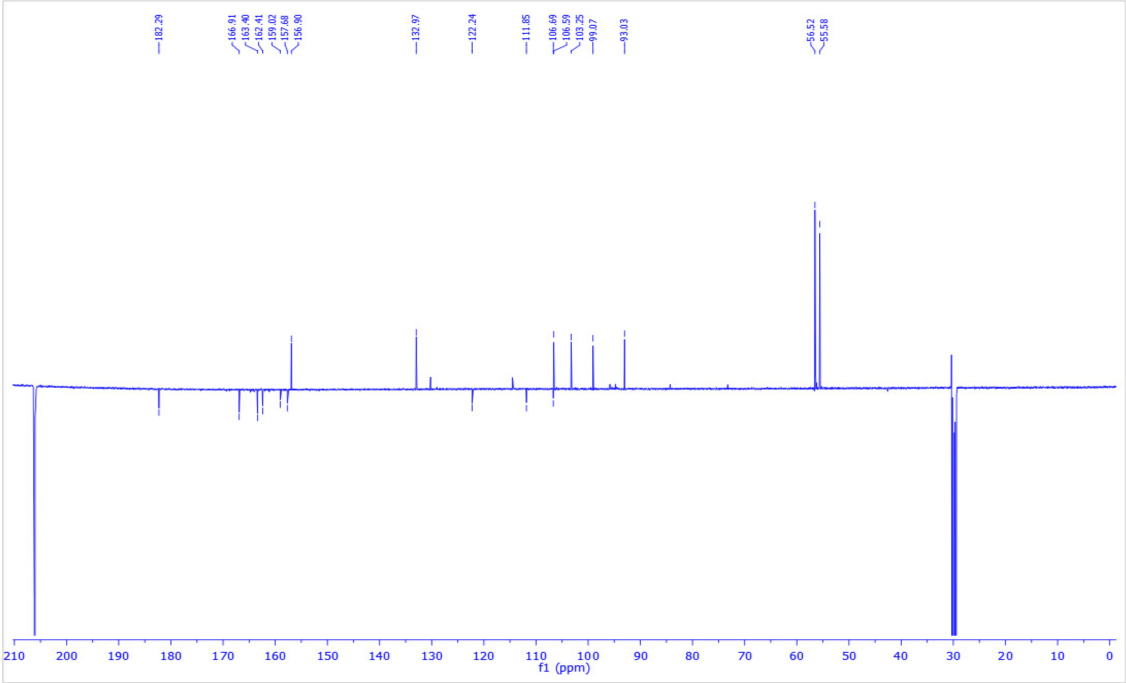

2',5-dihydroxy-4',7-dimethoxyflavanone (artocarpanone A)

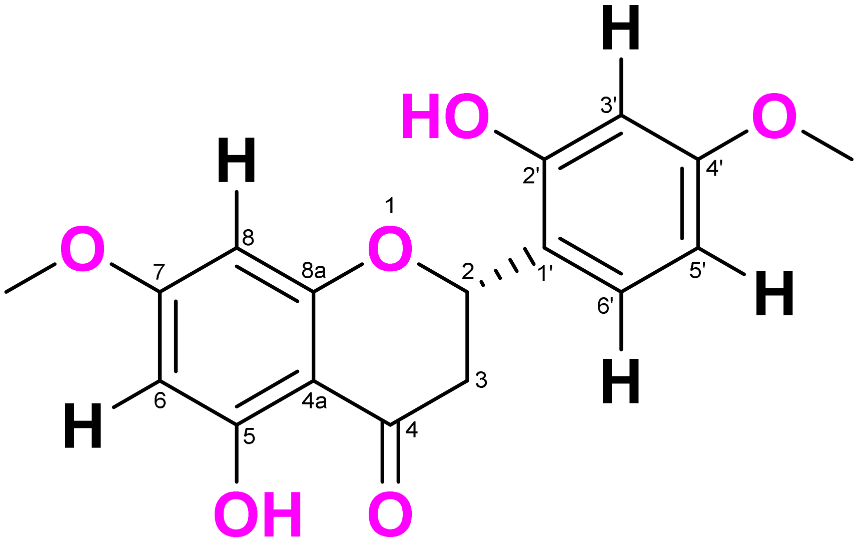

Table 4: ^{1}H, ^{13}C NMR spectroscopic data obtained for 2',5-dihydroxy-4',7-dimethoxyflavanone in Acetone-d_6, 600 and 150 MHz. Spectra were previously reported[3].

Position	δ_C, Type	δ_H, mult. (J in Hz)
**2**	75.4, CH	5.75, *dd* (13.1, 2.9)
**3ax**	42.6, CH_2	3.21, *dd* (17.2, 13.1)
**3eq**		2.77, *dd* (17.1, 3)
**4**	198.1, Cq	-
**4a**	104.3, Cq	-
**5**	165.1, Cq	-
**6**	95.4, CH	6.04, *d* (2.3)
**7**	168.8, Cq	-
**8**	94.5, CH	6.06, *d* (2.2)
**8a**	164.6, Cq	-
**1'**	118.5, Cq	-
**2'**	156.5, Cq HMBC	-
**3'**	102.3, CH	6.53, *d* (2.3)
**4'**	161.9, CH	-
**5'**	106.0, CH	6.54, *dd* (8.2, 1.9)
**6'**	129.0, CH	7.42, br *d* (8.8)
**7-OMe**	56.2, CH_3	3.85, s
**4'-OMe**	55.6, CH_3	3.77, s
**5-OH**	-	12.16, s
**2'-OH**	-	8.83, br s

Figure 21: Key ^{1}H-^{1}H COSY (**bold blue**), ^{1}H-^{13}C HMBC (H→C, red arrows) and ^{1}H-^{1}H ROESY (H↔H, green) of 2',5-dihydroxy-4',7-dimethoxyflavanone

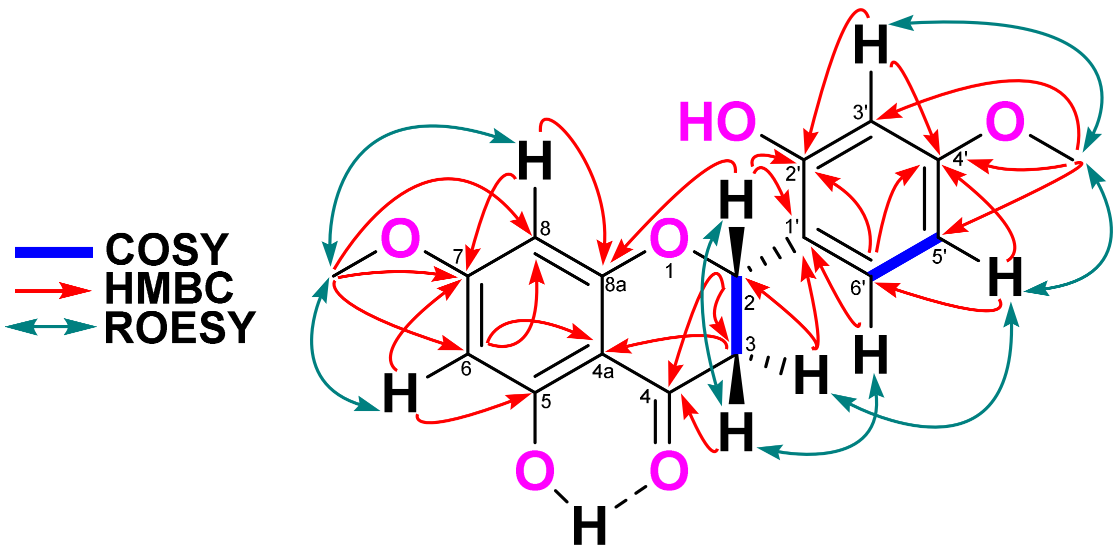

Figure 22: ^{1}H-NMR of 2',5-dihydroxy-4',7-dimethoxyflavanone in Acetone-d_6, 600 MHz

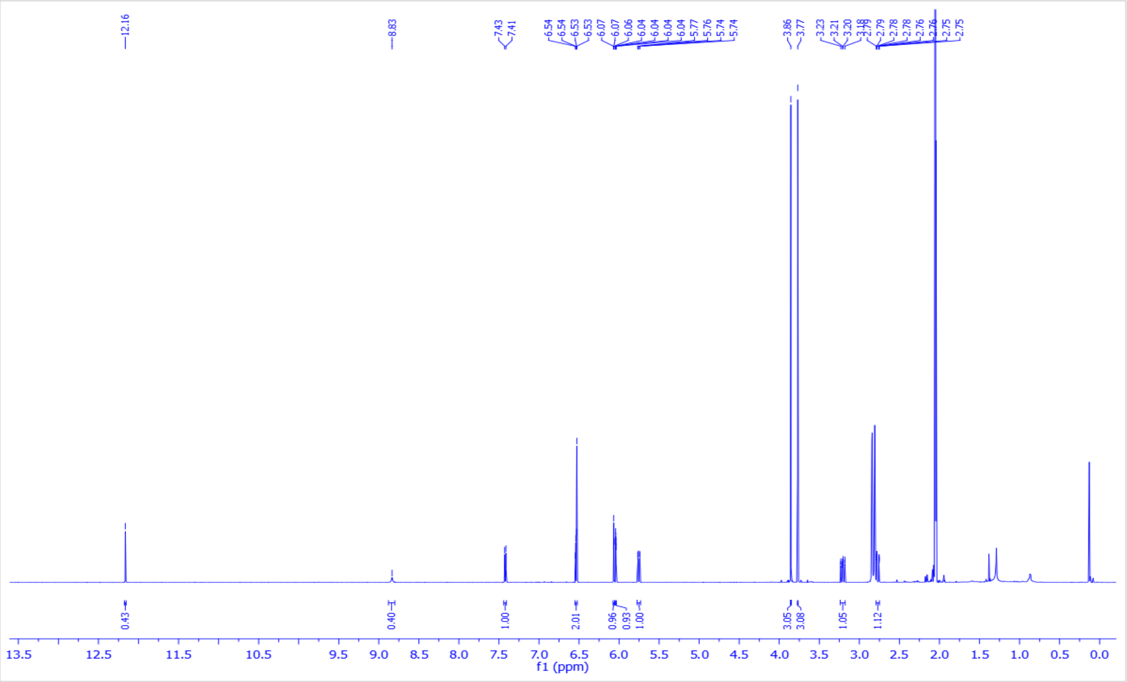

Figure 23: ^{1}H-^{1}H COSY of 2',5-dihydroxy-4',7-dimethoxyflavanone in Acetone-d_6, 600 MHz

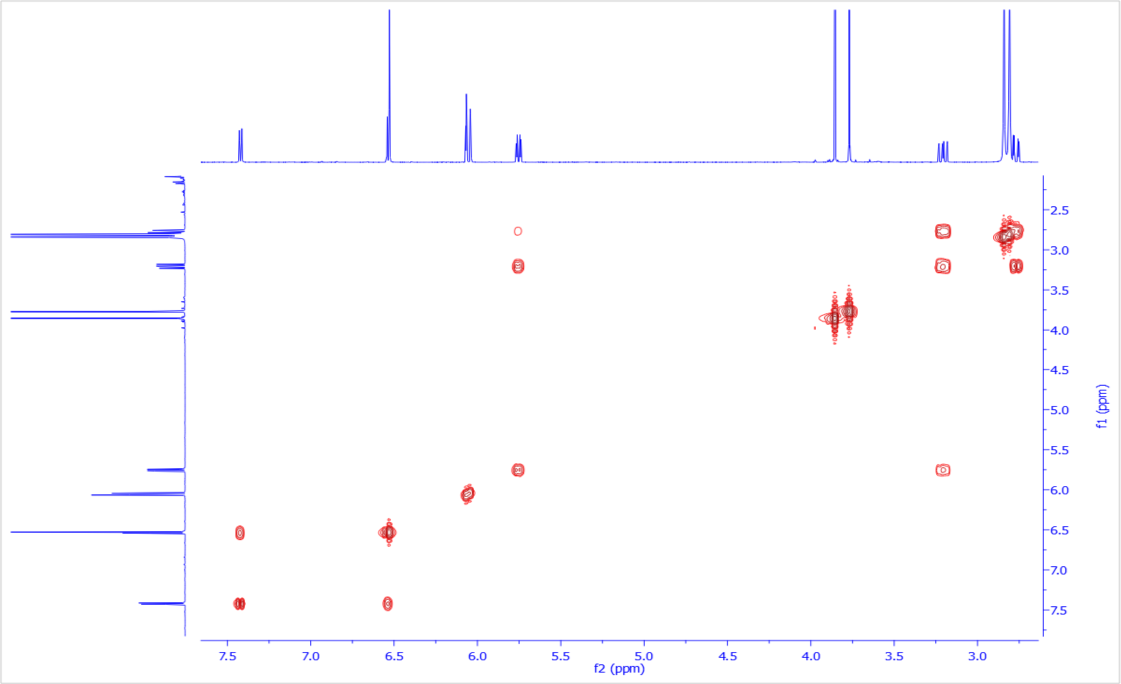

Figure 24: ^{1}H-^{13}C HSQC of 2',5-dihydroxy-4',7-dimethoxyflavanone in Acetone-d_6, 600 and 150 MHz

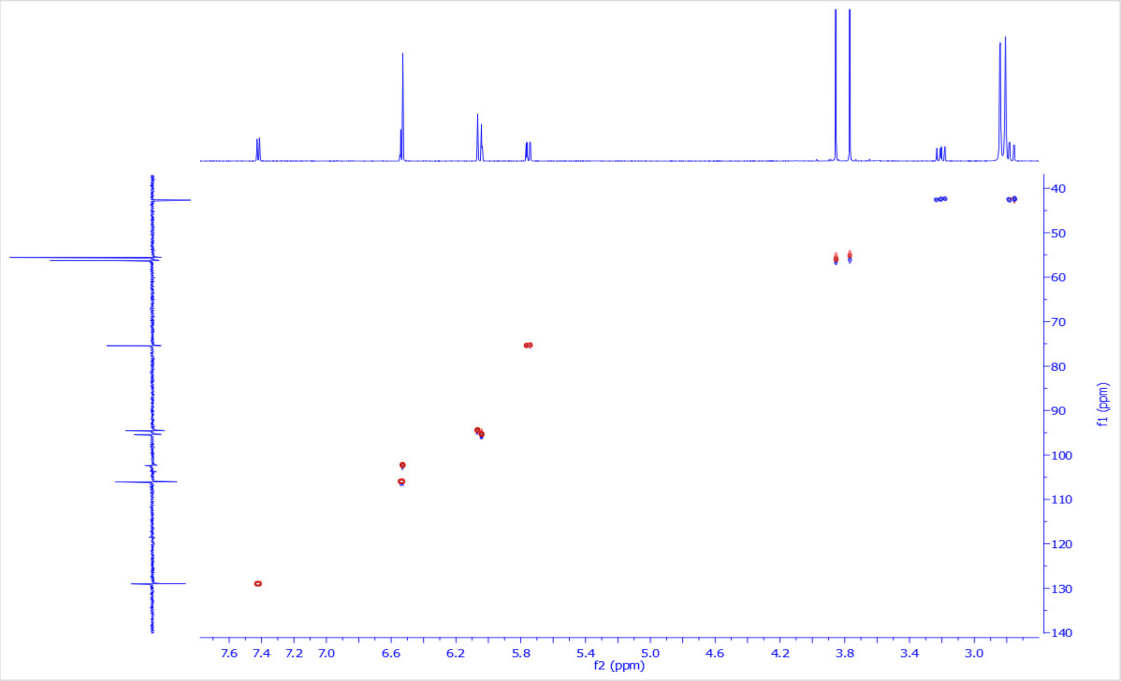

Figure 25: ^{1}H-^{13}C HMBC of 2',5-dihydroxy-4',7-dimethoxyflavanone in Acetone-d_6, 600 and 150 MHz

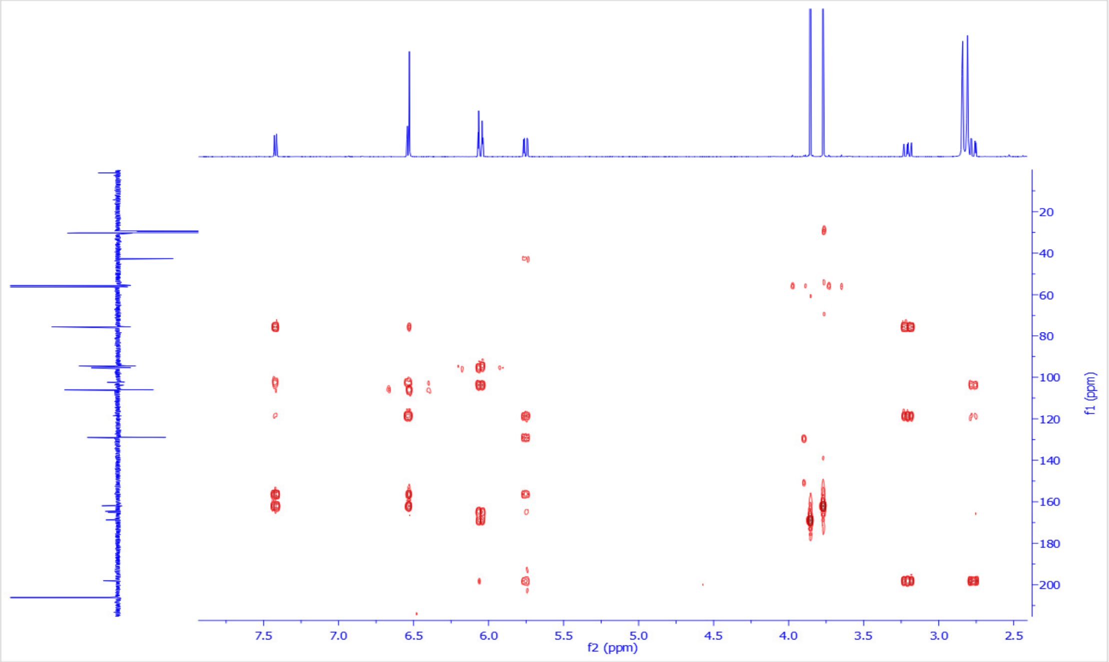

Figure 26: ^{1}H-^{1}H ROESY of 2',5-dihydroxy-4',7-dimethoxyflavanone in Acetone-d_6, 600 MHz

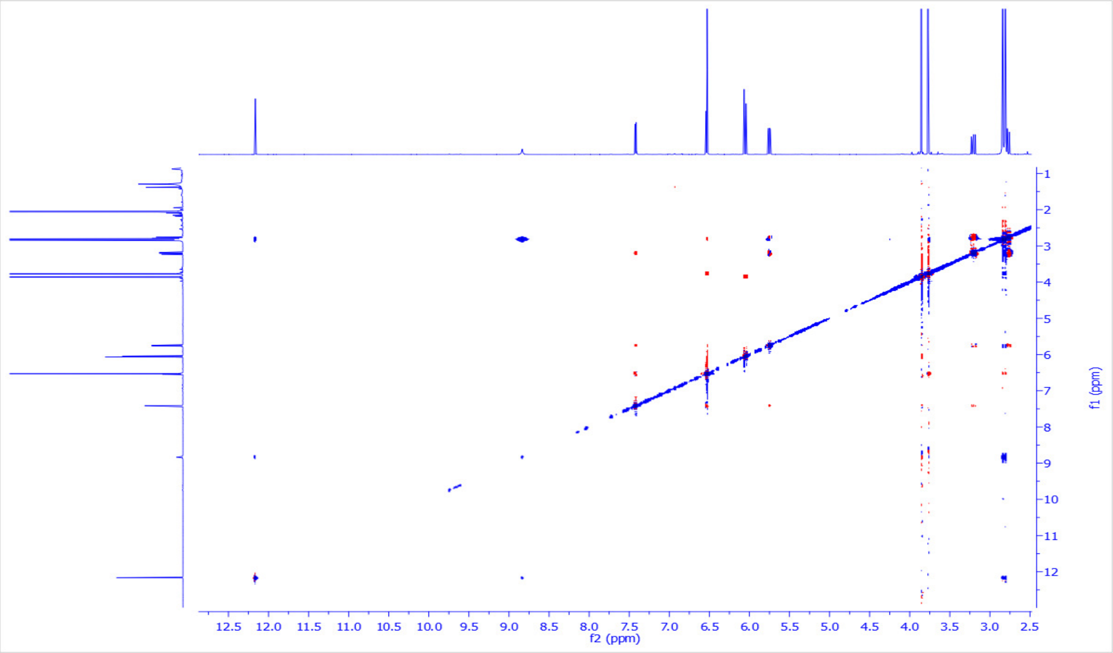

Figure 27: DEPTQ-135 of 2',5-dihydroxy-4',7-dimethoxyflavanone in Acetone-d_6, 150 MHz

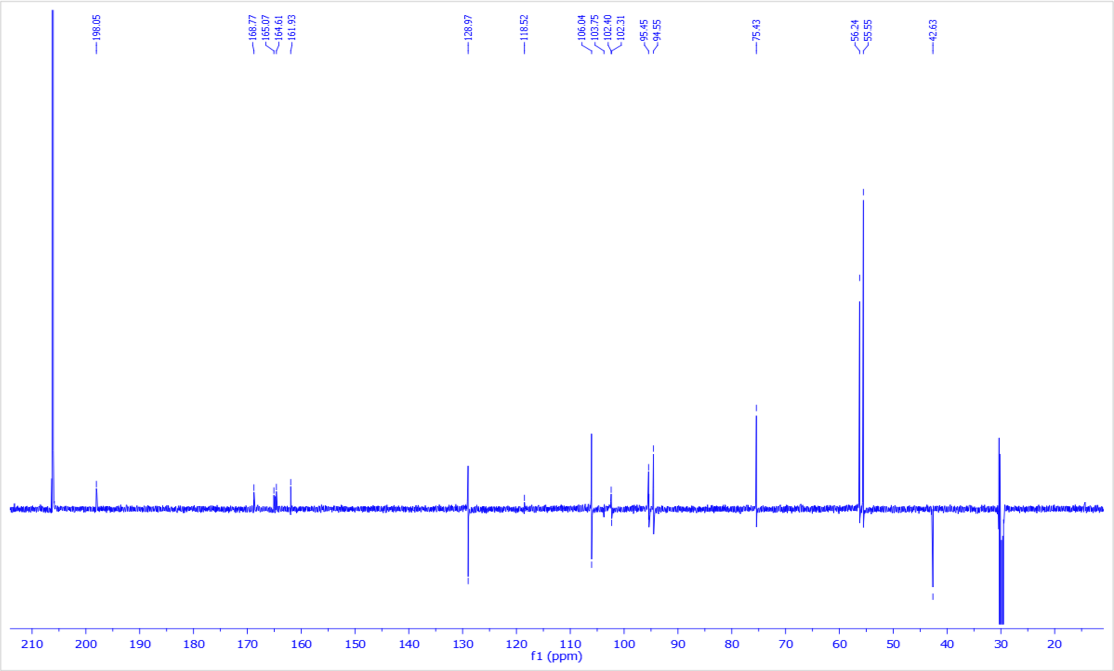

REFERENCES

1 Braz Filho, R., Pedreira, G., Gottlieb, O. R. & Maia, J. G. S. Chemistry of Brazilian Myristicaceae .8. Isoflavones from Virola-Caducifolia. *Phytochemistry* **15**, 1029-1030 (1976).

2 Islam, M. T. & Tahara, S. Dihydroflavonols from Lannea coromandelica. *Phytochemistry* **54**, 901-907 (2000).

3 Lin, C. N., Lu, C. M. & Huang, P. L. Flavonoids from Artocarpus-Heterophyllus. *Phytochemistry* **39**, 1447-1451 (1995).
